# The Impact of Imposing Equality Constraints on Residual Variances Across Classes in Regression Mixture Models

**DOI:** 10.3389/fpsyg.2021.736132

**Published:** 2022-01-27

**Authors:** Jeongwon Choi, Sehee Hong

**Affiliations:** Department of Education, Korea University, Seoul, South Korea

**Keywords:** regression mixture model, residual variance, equality constraint, parameter estimation, Monte Carlo simulation study

## Abstract

The purpose of this study is to explore the impact of constraining class-specific residual variances to be equal by examining and comparing the parameter estimation of a free model and a constrained model under various conditions. A Monte Carlo simulation study was conducted under several conditions, including the number of predictors, class-specific intercepts, sample size, class-specific regression weights, and class proportion to evaluate the results for parameter estimation of the free model and the restricted model. The free model yielded a more accurate estimation than the restricted model for most of the conditions, but the accuracy of the free model estimation was impacted by the number of predictors, sample size, the disparity in the magnitude of class-specific slopes and intercepts, and class proportion. When equality constraints were imposed in residual variance discrepant conditions, the parameter estimates showed substantial inaccuracy for slopes, intercepts, and residual variances, especially for those in Class 2 (with a lower class-specific slope). When the residual variances were equal between the classes, the restricted model showed better performance under some conditions.

## Introduction

Regression mixture modeling (RMM), which is a specific type of finite mixture modeling that detects latent classes within a population based on the difference in the relationship between a predictor and an outcome, is increasingly used in educational and behavioral research fields. Individuals that share the same regression function are clustered into the same latent class which shares a common regression function that is distinct to other latent classes. RMMs can be a flexible approach for detecting the heterogeneity of effects in situations when moderating variables are hard to identify or specify before designing potential research (Van Horn et al., 2012, 2015). However, RMMs have not been actively utilized until recently. One of the reasons for this dearth of research is that only a few simulation studies on RMMs have been conducted, so the characteristics of RMMs have not been thoroughly investigated. Furthermore, existing simulation studies on RMMs report its sensitivity to violating model assumptions and model misspecification that often result in unstable parameter estimates or non-convergence (Kim et al., 2016; Lamont et al., 2016; Wadsworth et al., 2018; Sherlock et al., 2021). However, issues in the performance of RMMs in parameter estimation, which is mainly of interest in mixture models, have not been thoroughly examined under various conditions yet. To be specific, as study designs, such as class proportion and class separation, are known to impact performance significantly (Jaki et al., 2019; Sherlock et al., 2021), this issue has not been addressed thoroughly, thus requiring more examination. Therefore, further simulation studies under more practical conditions and model specifications are necessary to uncover the performance of RMMs and provide guidelines for researchers.

In addition to simulation studies on the estimation of RMMs in general, the common practice in mixture modeling to constrain class-specific residual variances equally across classes for model parsimony also has not yet been thoroughly examined in the context of RMMs. Although mixture models suppose that residual variances can be different for each class, it is common to constrain them in empirical studies (Rights and Sterba, 2016) when researchers face problems in estimation. As such, the main reason that researchers impose the equality constraint on residual variances is for the estimation stability that can be described as follows (Enders and Tofighi, 2008). When those using mixture modeling encounter problems of model convergence, they often go through an exploratory process that constrains or equalizes some parameters that are not the main area of concern. If the constraints impact the performance of model estimation to a slight degree, it will be more parsimonious to constrain the parameters and focus on the other parameters that are the focus of the analysis. Accordingly, because class-specific residual variances are typically not interpreted or of interest in mixture models, they are often selected as the parameter to be constrained.

However, recommendations on constraining residual variances to be equal across classes in mixture modeling are inconsistent and inadequate. While some studies recommend constraining residual variances for model parsimony (Muthén and Asparouhov, 2009), some simulation studies report the high risk of these constraints when imposed without consideration (Enders and Tofighi, 2008; Kim et al., 2016). This issue should be discussed in detail particularly in RMM contexts because the impact has not been thoroughly understood yet, but the constraint strategy would be of interest to researchers using RMMs when facing problems in model convergence or estimation. As RMMs are known to require large sample sizes for stable estimation because there are many parameters to be estimated, these problems are common (Van Horn et al., 2015). Thus, this issue can be relevant to researchers using RMMs with a small sample as a critical solution for model estimation.

Although an existing study on this issue in RMMs implied that equally constraining residual variances across classes may result in unreliable parameter estimation (Kim et al., 2016), this issue has not been explored under various research settings that applied researchers may often encounter. Considering that conducting RMMs with a small sample size could yield problematic results even when correctly specified, the impact of the constraint that leads to misspecification would lead to different results from cases with large sample sizes. Therefore, it is necessary to expand on the previous work of Kim et al. (2016) to provide guidelines for the application of RMMs in realistic conditions.

As such, the purpose of this study is to compare a freely estimating model and a restricted model, which differ in model specification regarding residual variances under somewhat harsh conditions that are often found in applied study settings. With this simulation study, not only the performance in general of RMMs but also the issue of these constraints on residual variances can be addressed in detail. The research questions are as follows. First, under which conditions does the free model estimate the parameters accurately? Second, under which conditions does the restricted model estimate the parameters accurately? Third, under which conditions does the restricted model more accurately estimate the parameters than the free model?

## Theoretical Background

**FIGURE 1:**
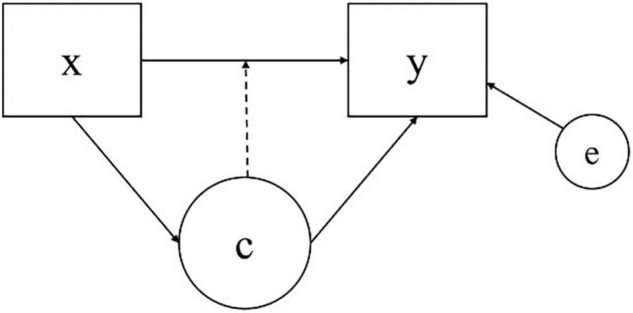
Diagram of an regression mixture modeling (RMM).

Regression mixture model is a specific type of finite mixture modeling that captures unobserved heterogeneity in the relationship between predictor(s) and an outcome that is present in a population. Based on the concept of regression modeling, which attempts to reveal the relationship between an independent variable and a dependent variable, RMM is used to explore unobserved subgroups in a population based on the difference in the effects of a predictor on an outcome in the framework of mixture modeling. Unlike other mixture models such as latent class models or growth mixture models that distinguish distinct latent classes with the differences in means or variances of outcomes of each class, RMMs capture heterogeneity that exists in data based on the relationship between x and y, which is quantified as a class-specific regression weight and intercept. A categorical latent class variable is incorporated in a typical regression model to capture the heterogeneity in the regression relationship between a predictor and an outcome within a population, where the latent class variable is measured by the conditional distribution of an outcome variable regressed on a predictor variable (Masyn, 2013). A model diagram of RMM is described in Figure 1, where, x denotes the predictor, y the outcome, e the residual, and C the latent class variable.

The assumptions on which RMMs rely can be described as follows (Wadsworth et al., 2018). First, for all *X*, $V\,a\,r\,\left(X,k\right)=\sigma_{k}^{2}$, variance within a class is homogenous among the individuals. Second, $\varepsilon_{i\,k}∼N\,\left(0,\sigma_{k}^{2}\right)$, class-specific residual variances differ across classes but are homogenous within a class. Third, $E\left(Y|X,k\right)=\beta_{0\,k}+\sum_{p=1}^{P}\beta_{p\,k}X_{i\,p}$, individuals within the same class share a common linear relationship between a predictor and an outcome. Previous studies showed that a violation of the assumptions in RMMs could disturb the precise estimation of parameters (Van Horn et al., 2012; George et al., 2013a; Kim et al., 2016).

Moreover, previous simulation studies on RMMs report sensitivity to model assumptions and specifications that quite rigorous conditions of data are required for stable estimation. These traits of RMMs require more exploration of the model performance under various circumstances to understand what the acceptable conditions are for reliable estimations and problematic results obtained with poor-quality estimation, which is crucial for an appropriate examination in any given study. Jaki et al. (2019) investigated the performance of RMMs under various conditions focusing on the impact of sample size. In most cases, a large sample and high class separation are required for satisfactory estimation results, and the results are better when an additional predictor is included in the model. A sample size under 1,000 can lead to problematic estimation results when class separation is not high enough. Meanwhile, there have been a few issues regarding the estimation of parameters in bias and coverage. To begin with, the biasedness of the parameters differed between two classes. That is, for some harsh conditions, while parameters for a class with a larger regression slope were well estimated, another class with a smaller regression slope resulted in a severe upward bias in intercepts and a downward bias in regression weights and residual variances. Next, coverages were slightly less than the desirable value even under good conditions with large samples or with substantial class separation. Even when the parameters were fairly estimated to be close to the true value under some small sample size conditions, the 95% coverage rates were poor. George et al. (2013a) showed that the violation of the normality assumption could lead to unreliable parameter estimates. Lamont et al. (2016) examined parameter estimation results when the path from a latent class variable to a predictor (i.e., C on *x* path) was excluded in two-class RMMs, which constrained class-specific predictor means to be equal. Inaccurate parameter estimates are obtained inaccurately when the mean of the predictors of each class was constrained to be equal although the sizes were different. Under the different-mean conditions, although the bias in parameter estimates was negligible in some conditions, it always resulted in biased class proportion and poor coverage rates. Specifically, the residual variance in a class with a smaller regression weight was inaccurately obtained in terms of the bias, RMSE (Root Mean Square Error), and coverage.

A typical strategy in mixture modeling in solving convergence problems is to decrease the number of parameters so that class-specific residual variances can be accordingly constrained to be equal. Related to this issue, whether constraining class-specific residual variances is appropriate or not has remained a controversy in finite mixture models. Although some literature on mixture modeling suggests that researchers constrain residual variances to be equal for model parsimony (Muthén and Asparouhov, 2009), the impact of this constraint has not been examined thoroughly in various situations although it can be expected to produce serious problems due to model misspecification. Enders and Tofighi (2008) studied the impact of constraining class-specific residual variances in growth mixture models, and the results showed that the large difference in class-specific residual variances, unequal class proportion, and more class-specific residual variance in the population produce serious bias in the estimates of within-class growth trajectories and variance components. Kim et al. (2016) attempted to figure out the consequences of constraining class-specific residual variances equally across classes in a relatively simple context. To summarize the results, the selection rate for the correct number of classes decreased significantly when equality constraints were imposed in residual variances while the disparity in the magnitude of residual variances was large. For parameter estimation, there was a severe bias in regression weights and class proportion when residual variances were inappropriately constrained. When constraints on residual variances were imposed even the difference in residual variances was large, the regression coefficients showed substantial bias that were downward biased or showed different signs in slopes with smaller effects because parameters, especially for Class 1, had large variation and extreme solutions. The bias and the RMSE were low, and coverage rates were mostly over 90% when the class-specific residual variances were the same.

Although the study of Kim et al. (2016) attempted to examine the issue of RMMs, still, there were some limitations that require further study. First, this study was conducted under very ideal conditions where the sample size was set to be considerably large and only balanced class proportion was considered in the study and did not reflect realistic conditions that are often encountered in applied research settings. Second, the impact of the disparity in the magnitude of class-specific intercepts between the classes, which is a crucial factor in manipulating class separation (George et al., 2013b), was not examined in a single-predictor model. Third, the model specifications regarding the residual variances were different between the univariate and the multivariate models; thus, more studies are needed for an intuitive comparison.

## Methods

In order to explore the impact of imposing equality constraints on class-specific residual variances in RMMs, Monte Carlo simulation studies were conducted. Two-class models were employed for the analysis in order to keep the context simple to clearly examine the consequence of constraining residual variances to be equal.

Four kinds of models were used for analysis: (1) a single-predictor model with no intercept difference, (2) a single-predictor model with an intercept difference of 1, (3) a two-predictor model with no intercept difference, and (4) a two-predictor model with an intercept difference of 1. These models followed the general model specification described by Kim et al. (2016) to effectively compare the models’ performances with the results from cases with larger sample sizes (i.e., sample size of 3,000). We additionally used single-predictor models with differences in class-specific intercepts to closely examine the impact of class separation size on the accuracy of parameter estimates, given the observation from Kim et al. (2016) that the models will perform better in conditions with greater class separation as obtained with greater differences in class-specific intercepts. As we aimed to examine the performances of the models with smaller sample sizes (which would require harsher conditions for the models to perform accurately), examining class separation was necessary for coming to a more instructive conclusion. One hundred replications were generated for each condition and the generated data were fit twice to the models; one was a model with freely estimated class-specific residual variances, and the other was a model estimated with equal constraints on the residual variances. Throughout the study, the simulated data were generated and analyzed using Mplus 8.3 (Muthén and Muthén, 1998–2017), and MplusAutomation package in R (Hallquist and Wiley, 2018) was used to run the models repeatedly.

For the single-predictor models, class-specific intercepts were manipulated to differ. One was set to be 0 in class-specific intercepts, and the other was set to have values in intercept 1 in Class 1 and 0 in Class 2. The former model was named “a single-predictor model with no intercept difference,” and the latter model was called “a single-predictor model with intercept difference 1.” A predictor *X* was drawn from normal distribution with mean 0 and variance 1, that is *X*∼*N*(0,1). Second, the class-specific regression weights *a*_1_ and *a*_2_ were set to accommodate both the magnitude of class separation and difference in class-specific residual variances as the value of class-specific residual variance can be obtained using the formula *Var*(*Y*) = *Var*(*X*) + *Var*(*e*) and the total variance of *Y* to be 1 in each class. Three sets of slope values were considered in the current study: 0.7 and 0.2, 0.7 and 0.4, and 0.5 and −0.5 for *a*_1_ and *a*_2_, respectively. These values were set based on previous simulation studies on regression mixtures to enable intuitive examination and comparison with previous studies. The first set included conditions for high class separation, but considered minimum values required for RMMs to effectively identify differential effects in previous studies (Lamont et al., 2016; Jaki et al., 2019; Kim et al., 2019) and the values in the second set are often set in RMMs to seek the consequences of conditions with low class separation (Jaki et al., 2019; Kim et al., 2019). The third condition, set to only differ in its sign, was considered to reflect situations where class-specific residual variances are equal and to examine the consequences of constraining residual variances when they have equal value (Kim et al., 2016).

Class-specific residual variances for each set of slopes were calculated using the formula given before, and the disparity in the magnitude of residual variances between classes was drawn. The three conditions are named “large-difference,” “moderate-difference,” and “non-difference” conditions by the magnitude of the residual variance difference between the two classes. In addition to the single-predictor models, multivariate models with two predictors are considered. Similar to the single-predictor models, class-specific intercepts are manipulated: two-predictor models with intercept zero in both classes can be named “two-predictor model with no intercept difference,” and “two-predictor models with difference in class-specific intercepts.” Class 1 having intercept 1 and Class 2 having 2 can be named “two-predictor model with intercept difference 1.” As can be seen in the equations above, the coefficients of each predictor within a class were set to be the same following the previous simulation design in multivariate RMMs (Kim et al., 2016; Jaki et al., 2019). Predictors *X*_1_ and *X*_2_ were drawn from normal distributions with mean 0 and variance 1, and the correlation between *X*_1_ and *X*_2_ was set to be 0.5. The residuals *e* were set to be normally distributed with mean 0, and variance was calculated using the formula given beforehand. Although the values for the coefficients of predictors were taken from the single-predictor models, the values were adjusted by taking the correlation between the predictors into consideration. The details of the model specifications are given in Table 1.

**TABLE 1:** Model specifications.

		Slope	Intercept	Residual variance
Single-predictor model with no intercept difference	Large	0.7/0.2	0/0	0.51/0.96
	Moderate	0.7/0.4		0.51/0.84
	No	0.5/−0.5		0.75/0.75

Single-predictor model with intercept difference 1	Large	0.7/0.2	1/0	0.51/0.96
	Moderate	0.7/0.4		0.51/0.84
	No	0.5/−0.5		0.75/0.75

Two-predictor model with no intercept difference	Large	0.6261/0.17889	0/0	0.02/0.92
	Moderate	0.6261/0.35777		0.02/0.68
	No	0.44721/−0.44721		0.5/0.5

Two-predictor model with intercept difference 1	Large	0.6261/0.17889	1/0	0.02/0.92
	Moderate	0.6261/0.35777		0.02/0.68
	No	0.44721/−0.44721		0.5/0.5

For the models described above, sample size and the proportion of individuals in each class were also manipulated. First, for sample size, four levels were set: 300, 500, 1,000, and 2,000. Although some previous simulation studies on RMMs considered 3,000 or 6,000 as a sufficient sample size for a stable estimation (George et al., 2013a; Kim et al., 2016), as this study attempts to explore its performance of models under realistic conditions, smaller sample sizes that can be commonly found in empirical research were considered to address the limitations of previous studies. Second, two different class proportions were considered in the present study: 50% in each class for a balanced class proportion, 75% in Class 1, and 25% in Class 2 for an unbalanced class proportion, following the studies of Jaki et al. (2019) and Sherlock et al. (2021).

To summarize, for the four models given above, three slope conditions, four sample size conditions, and two class proportion conditions were manipulated. Thus, for 4 × 3 × 4 × 2 = 96 conditions, 96 × 100 = 9,600 datasets are generated. The replications generated for each condition were fitted twice to two models. One was a model that freely estimates class-specific residual variances, and the other was a model that constrains class-specific residual variances to be equal across classes. Maximum likelihood estimation with robust standard errors (MLR) was used for estimation.

To evaluate the accuracy in the estimation of models with freely-estimated residual variance in each class and the constrained models where the residual variances are estimated to be the same within a class, each simulation condition is evaluated using three criteria: parameter bias, mean squared error (MSE), and coverage of a 95% confidence interval. Before the evaluation, convergence rates and parameter estimates were first examined to provide a basis for evaluating parameter accuracy.

## Results

### Convergence Rates

For single-predictor models and two-predictor models with sample sizes of 1,000 and 2,000, all replications for the freely estimated model and constrained model successfully converged. Although the single-predictor model converged under most conditions when residual variances are constrained, some of the replications in freely estimating models failed to converge, especially for moderate-difference conditions in a sample size of 300. These conditions can be considered low class separation because the slope difference between the two classes is the smallest. Similarly, for some conditions with unbalanced class proportion with a sample size of 500, some replications did not converge. Full results are given in Table 2.

**TABLE 2:** Convergence rates.

			SS = 300		SS = 500		SS = 1,000		SS = 2,000	
	CP	D_RV	FREE	CONS	FREE	CONS	FREE	CONS	FREE	CONS
**Single-predictor models**										
With no intercept difference	0.5:0.5	Large	1.00	1.00	1.00	1.00	1.00	1.00	1.00	1.00
		Moderate	0.99	1.00	1.00	1.00	1.00	1.00	1.00	1.00
		No	1.00	1.00	1.00	1.00	1.00	1.00	1.00	1.00
	0.75:0.25	Large	1.00	1.00	0.98	1.00	1.00	1.00	1.00	1.00
		Moderate	0.97	1.00	0.99	1.00	1.00	1.00	1.00	1.00
		No	1.00	1.00	1.00	1.00	1.00	1.00	1.00	1.00
With intercept difference 1	0.5:0.5	Large	1.00	1.00	1.00	1.00	1.00	1.00	1.00	0.96
		Moderate	0.99	1.00	1.00	1.00	1.00	1.00	1.00	1.00
		No	1.00	1.00	1.00	1.00	1.00	1.00	1.00	1.00
	0.75:0.25	Large	1.00	1.00	1.00	1.00	1.00	1.00	1.00	1.00
		Moderate	0.99	1.00	1.00	1.00	1.00	1.00	1.00	1.00
		No	1.00	1.00	1.00	1.00	1.00	1.00	1.00	1.00
**Two-predictor models**										
With no intercept difference	0.5:0.5	Large	1.00	1.00	1.00	1.00	1.00	1.00	1.00	1.00
		Moderate	1.00	1.00	1.00	1.00	1.00	1.00	1.00	1.00
		No	1.00	1.00	1.00	1.00	1.00	1.00	1.00	1.00
	0.75:0.25	Large	1.00	1.00	1.00	1.00	1.00	1.00	1.00	1.00
		Moderate	1.00	1.00	1.00	1.00	1.00	1.00	1.00	1.00
		No	1.00	1.00	1.00	1.00	1.00	1.00	1.00	1.00
With intercept difference 1	0.5:0.5	Large	1.00	1.00	1.00	1.00	1.00	1.00	1.00	1.00
		Moderate	1.00	1.00	1.00	1.00	1.00	1.00	1.00	1.00
		No	1.00	1.00	1.00	1.00	1.00	1.00	1.00	1.00
	0.75:0.25	Large	1.00	1.00	1.00	1.00	1.00	1.00	1.00	1.00
		Moderate	1.00	1.00	1.00	1.00	1.00	1.00	1.00	1.00
		No	1.00	1.00	1.00	1.00	1.00	1.00	1.00	1.00

*FREE, residual variances freely estimated, CONS, residual variances equally constrained, SS, sample size, CP, class proportion, D_RV, the disparity in the magnitude of class-specific residual variances.*

### Parameter Estimates

The parameter estimates of slope(s), intercept, and residual variance for each latent class are reported in Tables 3–6. The results for single and two-predictor models are similar in general, but the estimates of two-predictor models are more accurately obtained. When the intercepts are different between two classes, the estimates are closer to the true values.

**TABLE 3:** Parameter estimates of single-predictor models with no intercept difference.

				CP = 0.5:0.5 (balanced)								CP = 0.75:0.25 (unbalanced)							
				SS = 300		SS = 500		SS = 1,000		SS = 2,000		SS = 300		SS = 500		SS = 1,000		SS = 2,000	
D_RV	C	PAR	TRUE	FREE	CONS	FREE	CONS	FREE	CONS	FREE	CONS	FREE	CONS	FREE	CONS	FREE	CONS	FREE	CONS
Large	1	S	0.7	0.6106	0.4420	0.6617	0.5181	0.6395	0.4728	0.6133	0.3802	0.6395	0.4728	0.6028	0.3802	0.6016	0.3821	0.5815	0.5330
		I	0	−0.1793	−0.6145	−0.1151	0.0193	−0.0018	0.0608	0.0011	−0.0041	−0.0018	0.0608	0.0207	−0.0041	−0.0227	0.0585	−0.0238	−0.0413
		RV	0.51	0.4352	0.6760	0.4889	0.6931	0.4961	0.7102	0.5625	0.7180	0.4961	0.7102	0.4920	0.7180	0.5412	0.6016	0.5990	0.6056
	2	S	0.2	0.0955	0.1999	0.1438	0.0943	0.1848	0.6414	0.2783	0.0474	0.1848	0.6414	0.1035	0.0474	0.1651	0.3418	0.2604	−0.0902
		I	0	−0.1480	0.1206	0.3512	0.0603	0.0632	0.0156	0.0337	−0.0086	0.0632	0.0156	0.0738	−0.0086	0.0902	0.1196	0.0025	0.1536
		RV	0.96	0.6067	0.6760	0.6410	0.6931	0.8319	0.7102	0.8558	0.7180	0.8319	0.7102	0.5276	0.7180	0.6871	0.6016	0.8297	0.6056
Moderate	1	S	0.7	0.5983	0.4105	0.5453	0.4857	0.7035	0.4706	0.6121	0.4480	0.7035	0.4706	0.6121	0.4480	0.6739	0.6899	0.6199	0.3826
		I	0	−0.0307	−0.0683	−0.1252	−0.0765	−0.0061	0.7694	−0.0474	−0.0883	−0.0061	0.7694	−0.0470	−0.0883	0.0503	−0.0591	−0.0256	−0.0217
		RV	0.51	0.4152	0.6107	0.4155	0.6283	0.4626	0.6452	0.5222	0.6673	0.4626	0.6452	0.5222	0.6673	0.3913	0.5651	0.4986	0.5763
	2	S	0.4	0.3121	0.6260	0.4188	0.6542	0.3318	0.5548	0.3842	0.2198	0.3318	0.5548	0.3842	0.2198	0.4300	0.3511	0.3917	0.4908
		I	0	−0.2165	−0.0522	0.3577	−0.2024	−0.0206	0.1004	0.0284	0.0498	−0.0206	0.1004	0.0284	0.0498	0.1976	0.2680	0.2191	0.0162
		RV	0.84	0.4637	0.6107	0.4926	0.6283	0.7907	0.6452	0.7619	0.6673	0.7907	0.6452	0.7619	0.6673	0.4802	0.5651	0.4974	0.5763
No	1	S	0.5	0.4638	0.3032	0.4461	0.4700	0.4230	0.4500	0.3745	0.4630	0.4230	0.4500	0.3745	0.4630	0.4611	0.3870	0.4218	0.3670
		I	0	−0.0476	0.3611	0.0012	0.0146	−0.0163	0.0055	−0.0057	0.0063	−0.0163	0.0055	−0.0060	0.0063	−0.0022	−0.0070	0.0031	−0.0090
		RV	0.75	0.6939	0.7364	0.7204	0.7359	0.7223	0.7408	0.7396	0.7470	0.7223	0.7408	0.7396	0.7470	0.7252	0.7386	0.7431	0.7467
	2	S	−0.5	−0.5364	−0.4521	−0.4899	−0.4714	−0.4363	−0.4326	−0.3933	−0.4603	−0.4363	−0.4326	−0.3930	−0.4603	−0.4928	−−0.3658	−0.4552	−0.3568
		I	0	0.0823	−0.0796	0.0453	−0.0023	0.0040	−0.0008	0.0042	−0.0028	0.0040	−0.0008	0.0042	−0.0028	0.0024	0.0065	−0.0012	0.0037
		RV	0.75	0.6327	0.7364	0.6791	0.7359	0.7400	0.7408	0.7432	0.7470	0.7400	0.7408	0.7432	0.7470	0.7054	0.7386	0.7254	0.7467

*FREE, residual variances freely estimated; CONS, residual variances equally constrained; SS, sample size; CP, class proportion; D_RV, the disparity in the magnitude of class-specific residual variances; C, latent class; TRUE, population values for each parameter; PAR, parameters; S, class-specific slope; I, class-specific intercept; RV, class-specific residual variance.*

**TABLE 4:** Parameter estimates of single-predictor models with intercept difference 1.

				CP = 0.5:0.5 (balanced)								CP = 0.75:0.25 (unbalanced)							
				SS = 300		SS = 500		SS = 1,000		SS = 2,000		SS = 300		SS = 500		SS = 1,000		SS = 2,000	
D_RV	C	PAR	TRUE	FREE	CONS	FREE	CONS	FREE	CONS	FREE	CONS	FREE	CONS	FREE	CONS	FREE	CONS	FREE	CONS
Large	1	S	0.7	0.6833	0.6833	0.6803	0.4269	0.6691	0.4445	0.6641	0.4466	0.6428	0.4654	0.6881	0.4421	0.6346	0.4707	0.6276	0.4374
		I	1	0.8677	0.8677	0.9650	0.6564	0.9669	0.6471	0.9564	0.6249	0.9705	0.6999	0.9213	0.6387	0.8649	0.7223	0.8450	0.6173
		RV	0.51	0.5086	0.5086	0.5126	0.6187	0.5132	0.6249	0.5245	0.6262	0.5241	0.5435	0.5188	0.5465	0.5580	0.5537	0.5547	0.5579
	2	S	0.2	0.2300	0.2300	0.2305	0.6004	0.2178	0.5900	0.2236	0.5849	0.3274	0.6320	0.2784	0.6339	0.2703	0.6386	0.2688	0.6435
		I	0	−0.2777	−0.2777	−0.2148	−0.5532	−0.0945	−0.5302	−0.0245	−0.4998	−0.5080	−0.6320	−0.3376	−0.5601	−0.0958	−0.5752	0.0350	−0.5272
		RV	0.96	0.6963	0.6963	0.8079	0.6187	0.8760	0.6249	0.8983	0.6262	0.5816	0.5435	0.7109	0.5465	0.7845	0.5537	0.8467	0.5579
Moderate	1	S	0.7	0.6784	0.6784	0.6983	0.5687	0.6873	0.5989	0.6942	0.5905	0.7120	0.6202	0.6877	0.6024	0.6879	0.6186	0.6751	0.6022
		I	1	0.9762	0.9762	0.9438	0.6083	0.9652	0.6208	0.9747	0.6153	0.9889	0.6942	0.9205	0.6521	0.9391	0.6950	0.9117	0.6248
		RV	0.51	0.4412	0.4412	0.5039	0.5614	0.5008	0.5729	0.5002	0.5755	0.4763	0.5164	0.5087	0.5225	0.5173	0.5326	0.5171	0.5370
	2	S	0.4	0.5239	0.5239	0.4437	0.6073	0.4263	0.6296	0.4143	0.6278	0.5527	0.6363	0.5168	0.6158	0.4427	0.6583	0.4332	0.6643
		I	0	−0.3741	−0.3741	−0.2848	−0.5794	−0.1939	−0.5637	−0.1297	−0.5616	−0.4103	−0.7020	−0.4224	−0.6499	−0.2153	−0.6298	−0.0647	−0.6007
		RV	0.84	0.6604	0.6604	0.6469	0.5614	0.7218	0.5729	0.7687	0.5755	0.5119	0.5164	0.5662	0.5225	0.7028	0.5326	0.7538	0.5370
No	1	S	0.5	0.4276	0.4276	0.4586	0.2461	0.4455	0.3835	0.4283	0.3875	0.4233	0.0956	0.4505	0.0021	0.4468	−0.0648	0.4381	−0.2758
		I	1	1.0555	1.0555	0.9999	−0.3600	0.9616	−0.4414	0.9437	−0.4433	0.9974	−0.2012	0.9444	−0.2406	0.9380	−0.2296	0.9348	−0.1362
		RV	0.75	0.6874	0.6874	0.7065	0.7472	0.7399	0.7482	0.7441	0.7517	0.7374	0.7357	0.7297	0.7371	0.7420	0.7336	0.7460	0.7506
	2	S	−0.5	−0.4320	−0.4320	−0.4484	−0.2522	−0.4575	−0.3913	−0.4398	−0.3906	−0.4699	−0.0596	−0.4119	0.0106	−0.4557	0.0465	−0.4491	0.2708
		I	0	−0.0435	−0.0435	0.0175	−0.1237	0.0219	−0.0556	0.0531	−0.0584	−0.0722	−0.1844	−0.0793	−0.2595	0.0121	−0.2851	0.0429	−0.3781
		RV	0.75	0.6842	0.6842	0.7357	0.7472	0.7401	0.7482	0.7467	0.7517	0.5537	0.7357	0.6689	0.7371	0.7167	0.7436	0.7358	0.7506

*FREE, residual variances freely estimated; CONS, residual variances equally constrained; SS, sample size; CP, class proportion; D_RV, the disparity in the magnitude of class-specific residual variances; C, latent class; TRUE, population values for each parameter; PAR, parameters; S, class-specific slope; I, class-specific intercept; RV, class-specific residual variance.*

**TABLE 5:** Parameter estimates of two-predictor models with no intercept difference.

				CP = 0.5:0.5 (balanced)								CP = 0.75:0.25 (unbalanced)							
				SS = 300		SS = 500		SS = 1,000		SS = 2,000		SS = 300		SS = 500		SS = 1,000		SS = 2,000	
D_RV	C	PAR	TRUE	FREE	CONS	FREE	CONS	FREE	CONS	FREE	CONS	FREE	CONS	FREE	CONS	FREE	CONS	FREE	CONS
Large	1	S1	0.626	0.6258	0.4147	0.6239	0.5253	0.6257	0.6333	0.6264	0.7326	0.5406	0.7242	0.5640	0.8691	0.5397	0.7879	0.5387	0.8725
		S2	0.626	0.6245	0.2205	0.6252	0.1242	0.6254	0.0016	0.6256	−0.0994	0.5353	−0.0838	0.5603	−0.2341	0.5355	−0.1581	0.5415	−0.2533
		I	0	0.0004	−0.0014	−0.0006	0.0034	−0.0008	0.0074	−0.0002	0.0022	−0.0049	−0.0070	−0.0070	−0.0062	0.0036	−0.0047	−0.0048	0.0004
		RV	0.02	0.0196	0.4246	0.0196	0.4120	0.0200	0.3919	0.0201	0.3855	0.1748	0.1804	0.1334	0.1879	0.1817	0.1877	0.1938	0.1887
	2	S1	0.179	0.1723	0.4889	0.1647	0.5517	0.1717	0.6060	0.1757	0.5560	0.2543	0.6828	0.2325	0.5701	0.2668	0.6417	0.2651	0.5634
		S2	0.179	0.1739	0.1412	0.1781	0.0993	0.1768	0.0363	0.1776	0.0802	0.2710	−0.0645	0.2442	0.0602	0.2676	−0.0254	0.2642	0.0629
		I	0	−0.0274	0.0324	−0.0205	0.0264	−0.0036	−0.0025	−0.0022	0.0004	−0.0073	0.0000	−0.0084	−0.0006	−0.0018	0.0062	0.0110	0.0021
		RV	0.92	0.9179	0.4246	0.9161	0.4120	0.9158	0.3919	0.9241	0.3855	0.7390	0.1804	0.7894	0.1879	0.7354	0.1877	0.7417	0.1887
Moderate	1	S1	0.626	0.6260	0.7446	0.6224	0.6917	0.6263	0.6711	0.6268	0.6563	0.5697	0.9222	0.4455	0.9111	0.5724	0.8896	0.5805	0.6798
		S2	0.626	0.6243	−0.0845	0.6222	−0.0234	0.6255	−0.0113	0.6263	−0.0005	0.5752	−0.2797	0.4448	−0.2609	0.5748	−0.2501	0.5802	−0.0201
		I	0	0.0017	−0.0303	−0.0012	0.0113	−0.0002	0.0057	−0.0001	0.0022	0.0042	0.0235	−0.0028	−0.0197	0.0016	0.0068	0.0039	0.0080
		RV	0.02	0.0199	0.3410	0.0258	0.3389	0.0201	0.3295	0.0202	0.3285	0.1313	0.1588	0.4471	0.1663	0.1462	0.1655	0.1331	0.1661
	2	S1	0.358	0.3520	0.5515	0.3526	0.6110	0.3509	0.7208	0.3561	0.7451	0.4082	0.5856	0.5352	0.6241	0.4096	0.6359	0.4038	0.8486
		S2	0.358	0.3539	0.1037	0.3614	0.0494	0.3572	−0.0676	0.3586	−0.0940	0.4053	0.0871	0.5338	0.0523	0.4046	0.0264	0.4037	−0.2103
		I	0	−0.0233	0.0494	−0.0149	0.0078	−0.0033	0.0019	−0.0030	0.0004	−0.0187	−0.0263	−0.0114	−0.0022	−0.0002	0.0034	0.0014	−0.0022
		RV	0.68	0.6856	0.3410	0.6757	0.3389	0.6808	0.3295	0.6834	0.3285	0.5560	0.1588	0.2439	0.1663	0.5399	0.1655	0.5647	0.1161
No	1	S1	0.447	0.3724	0.0130	0.3864	0.0354	0.4003	0.0473	0.4507	0.1589	0.4141	0.0287	0.4228	0.0495	0.3967	0.0931	0.4226	0.1380
		S2	0.447	0.3766	0.5073	0.4005	0.5140	0.3987	0.5122	0.4409	0.3693	0.4059	0.4715	0.4291	0.4627	0.4022	0.4771	0.4162	0.3314
		I	0	−0.0001	0.0024	−0.0011	0.0844	0.0107	0.0237	0.0023	0.0606	−0.0069	−0.0447	−0.0061	0.0774	0.0033	−0.0624	−0.0012	−0.0068
		RV	0.5	0.4816	0.6599	0.4888	0.6798	0.4962	0.6907	0.5011	0.7030	0.4971	0.6209	0.4989	0.6419	0.4979	0.6557	0.5027	0.6554
	2	S1	−0.447	−0.3768	−0.0724	−0.3932	−0.0574	−0.3993	−0.0904	−0.4456	−0.1446	−0.4298	0.0034	−0.4382	−0.1102	−0.3957	−0.1563	−0.4208	−0.2330
		S2	−0.447	−0.3767	0.4646	−0.3866	0.4441	−0.4010	0.4382	−0.4484	0.3925	−0.4038	0.4391	−0.4191	0.4384	−0.4117	0.4141	−0.4199	0.2411
		I	0	−0.0133	0.1639	−0.0148	0.0152	−0.0077	−0.0481	0.0004	−0.0690	−0.0131	−0.0179	−0.0117	−0.0814	−0.0045	−0.0280	0.0003	−0.0048
		RV	0.5	0.4859	0.6599	0.4926	0.6798	0.4971	0.6907	0.4981	0.7030	0.4637	0.6209	0.4704	0.6419	0.4869	0.6557	0.4942	0.6554

*FREE, residual variances freely estimated; CONS, residual variances equally constrained; SS, sample size; CP, class proportion; D_RV, the disparity in the magnitude of class-specific residual variances; C, latent class; TRUE, population values for each parameter; PAR, parameters; S1, class-specific slope for x1; S2, class-specific slope for x2; I, class-specific intercept; RV, class-specific residual variance.*

**TABLE 6:** Parameter estimates of two-predictor models with intercept difference 1.

				CP = 0.5:0.5 (balanced)								CP = 0.75:0.25 (unbalanced)							
				SS = 300		SS = 500		SS = 1,000		SS = 2,000		SS = 300		SS = 500		SS = 1,000		SS = 2,000	
D_RV	C	PAR	TRUE	FREE	CONS	FREE	CONS	FREE	CONS	FREE	CONS	FREE	CONS	FREE	CONS	FREE	CONS	FREE	CONS
Large	1	S1	0.626	0.6261	0.4561	0.6258	0.5007	0.6264	0.4481	0.6267	0.5516	0.6100	0.3552	0.5991	0.3568	0.5776	0.2771	0.6068	0.1901
		S2	0.626	0.6241	0.1876	0.6245	0.1397	0.6255	0.3092	0.6259	0.0727	0.6150	0.2805	0.6080	0.2806	0.5818	0.3420	0.6087	0.4267
		I	1	0.9997	0.2195	0.9986	0.1944	0.9993	0.5704	0.9993	0.0732	0.9686	0.0723	0.9443	0.0955	0.8987	0.1380	0.9591	0.0918
		RV	0.02	0.0196	0.3758	0.0198	0.3871	0.0199	0.3879	0.0201	0.3931	0.0458	0.1861	0.0600	0.1923	0.1033	0.1959	0.0559	0.1941
	2	S1	0.179	0.1655	0.9181	0.1575	0.8683	0.1699	1.0019	0.1733	0.7927	0.1676	1.2428	0.1767	1.2375	0.2221	1.2906	0.1915	1.3324
		S2	0.179	0.1814	−0.2864	0.1874	−0.2298	0.1807	−0.3421	0.1772	−0.1576	0.2064	−0.6285	0.2071	−0.6232	0.2229	−0.6814	0.1951	−0.7306
		I	0	−0.0214	−0.8745	−0.0157	−0.8140	−0.0026	−0.9894	−0.0041	−0.7301	0.0116	−1.1975	0.0310	−1.2161	0.0976	−1.2768	0.0407	−1.3256
		RV	0.92	0.9180	0.3758	0.9201	0.3871	0.9178	0.3879	0.9235	0.3931	0.8662	0.1861	0.8601	0.1923	0.8136	0.1959	0.8798	0.1941
Moderate	1	S1	0.626	0.6263	0.6869	0.6253	0.6783	0.6264	0.6658	0.6272	0.6426	0.6112	0.6507	0.5240	0.6676	0.6020	0.6658	0.6179	0.6657
		S2	0.626	0.6247	−0.0235	0.6254	−0.0062	0.6255	0.0029	0.6256	0.0230	0.6022	0.0184	0.5221	0.0129	0.6037	0.0029	0.6184	0.0047
		I	1	1.0011	0.3338	0.9999	0.4025	1.0001	0.3863	0.9997	0.4497	0.9244	0.3487	0.6217	0.3608	0.9093	0.3863	0.9682	0.4239
		RV	0.02	0.0196	0.2900	0.0196	0.2919	0.0198	0.2921	0.0201	0.2932	0.0612	0.1571	0.2484	0.1611	0.0799	0.2921	0.0395	0.1639
	2	S1	0.358	0.3518	1.0911	0.3469	1.1252	0.3530	1.1276	0.3567	1.1466	0.3654	1.3184	0.4427	1.3260	0.3778	1.1276	0.3622	1.3426
		S2	0.358	0.3585	−0.4655	0.3605	−0.5064	0.3561	−0.5119	0.3576	−0.5323	0.3863	0.7080	0.4650	−0.7178	0.3760	−0.5119	0.3650	−0.7399
		I	0	−0.0175	−0.9652	−0.0110	−1.0164	−0.0027	−1.0269	−0.0019	−1.0850	0.0543	−1.2660	0.3570	−1.2557	0.0874	−1.0269	0.0307	−1.3044
		RV	0.68	0.6789	0.2900	0.6840	0.2919	0.6808	0.2921	0.6857	0.2932	0.6130	0.1571	0.4386	0.1611	0.6044	0.2921	0.6587	1.3487
No	1	S1	0.447	0.4276	0.1611	0.4378	0.1642	0.4313	0.1328	0.4441	0.1270	0.4172	0.1836	0.4260	0.1414	0.3140	0.0357	0.3205	0.0115
		S2	0.447	0.4308	0.3397	0.4439	0.2973	0.4282	0.2331	0.4328	0.1931	0.4154	0.3281	0.4320	0.3144	0.3175	0.2728	0.3060	0.2506
		I	1	0.9929	−0.0843	0.9982	−0.1415	0.9924	−0.2593	0.9943	−0.2619	0.9672	−0.1218	0.9761	−0.1401	0.8493	−0.1965	0.8473	−0.1625
		RV	0.5	0.4690	0.6208	0.4701	0.6384	0.4849	0.6451	0.4965	0.6565	0.4871	0.5909	0.4888	0.6144	0.4978	0.6267	0.4974	0.6366
	2	S1	−0.447	−0.4291	−0.1625	−0.4394	−0.0771	−0.4195	−0.0999	−0.4321	−0.1189	−0.4240	−0.0167	−0.4334	−0.0554	−0.3015	−0.0836	−0.3093	−0.1177
		S2	−0.447	−0.4222	0.3166	−0.4226	0.3756	−0.4283	0.3431	−0.4397	0.3708	−0.4045	0.3090	−0.4096	0.3127	−0.3183	0.2571	−0.3106	0.2178
		I	0	0.0083	−0.1203	−0.0038	−0.1829	0.0137	−0.1188	0.0105	−0.0955	0.0072	−0.1680	0.0098	−0.2114	0.1486	−0.1382	0.1529	−0.1233
		RV	0.5	0.4876	0.6208	0.4980	0.6384	0.5012	0.6451	0.5024	0.6565	0.4644	0.5909	0.4894	0.6144	0.4961	0.6267	0.5031	0.0272

*FREE, residual variances freely estimated; CONS, residual variances equally constrained; SS, sample size; CP, class proportion; D_RV, the disparity in the magnitude of class-specific residual variances; C, latent class; TRUE, population values for each parameter; PAR, parameters; S1, class-specific slope for x1; S2, class-specific slope for x2; I, class-specific intercept; RV, class-specific residual variance.*

The detailed results can be described as follows. When the residual variances are freely estimated, the parameter estimates are close to the true value when the sample size was 1,000 or 2,000. When the sample size is smaller than 1,000, the estimates are generally more deviated from the true value. It is remarkable that the estimates are closer to the true value even under conditions with lower class separation and smaller sample sizes when intercepts are set to be different between the models.

On the other hand, when the residual variances are constrained, the estimates for the intercepts are not that severe, but those for the slopes are too extreme to represent the true value; the class with a larger slope for the population value is found to have a smaller estimate than the other class in some conditions. Especially for the large-difference conditions, the estimations are poor. For non-difference conditions, although some estimates are slightly lower than the absolute value of the true value in some conditions, the estimates are fairly close to the true values.

### Bias

**FIGURE 2:**
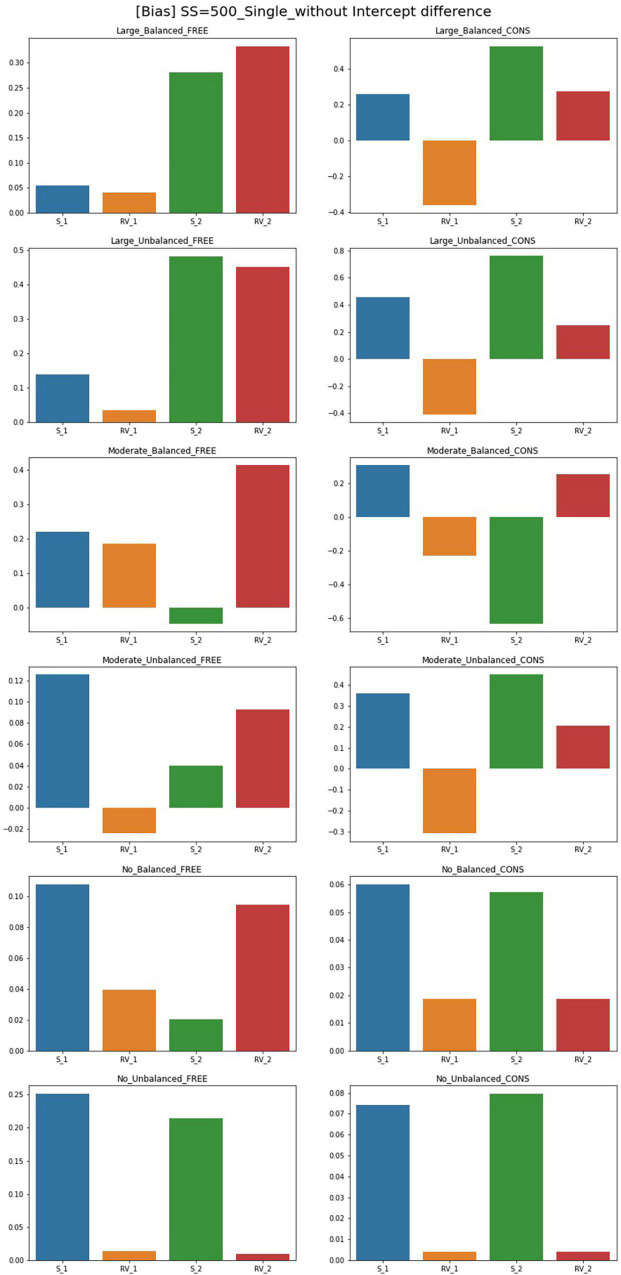
Parameter bias of single-predictor models (sample size = 500, without intercept difference). Free, residual variances freely estimated; Cons, residual variances equally constrained; S_1, slope for class 1; RV_1, residual variance for class 1; S_2, slope for class 2; RV_2, residual variance for class 2.

**FIGURE 3:**
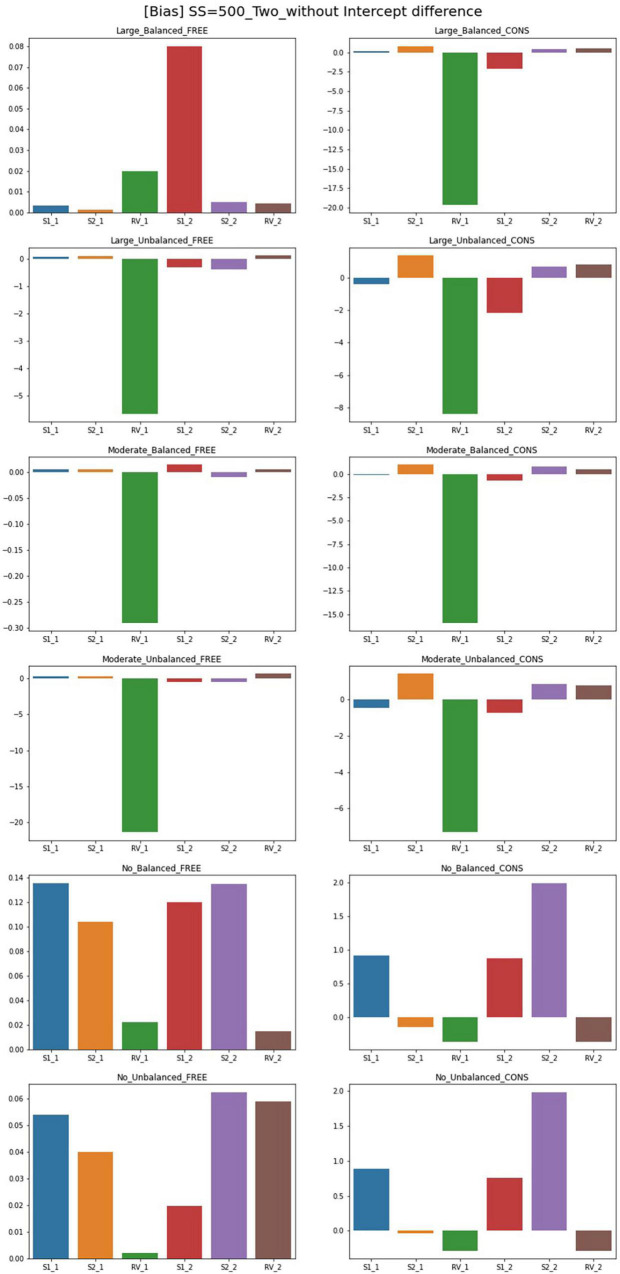
Parameter bias of two-predictor models (sample size = 500, without intercept difference). Free, residual variances freely estimated; Cons, residual variances equally constrained; S_1, slope for class 1; RV_1, residual variance for class 1; S_2, slope for class 2; RV_2, residual variance for class 2.

The results of the parameter bias for one and two predictor models with sample size 500 and intercept 0 are summarized in Figures 2, 3. The full tables and figures for bias, MSE, and coverage rates are available from the author upon request. The parameter bias shows the degree to which the parameter estimate deviates from the true population value. In this study, biases for the slope(s) and residual variance are calculated among the estimated parameters reported in the previous section. Since the intercept for Class 1 is under the conditions with an intercept difference, the denominator in the bias formula is zero. Therefore, the bias calculation for these intercepts is not available in this study. Instead, biasedness of the intercepts can be inferred from the results of the parameter estimates.

Although free models for some conditions show close parameter estimates to the true value, biases for most conditions exceed the cutoff point 0.1. For the single-predictor models, when intercepts are different between two classes, biases are low in general. With a larger sample size, there are more acceptable parameters where biases are lower than 0.1. For large-difference conditions with large sample sizes (SS = 1,000, SS = 2,000), the parameter biases are low under an intercept difference while some are high when the intercepts of the two classes are the same. Constrained models in large-difference conditions show an excessive bias for slope factor in Class 2, regardless of the existence of an intercept difference.

Under non-difference conditions, class proportion has a significant impact on the biases. For conditions with the same intercepts, constrained models under the balanced class proportion have lower biases except for the smallest sample size of 300. On the other hand, biases are generally high under the unbalanced settings, only being low for sample sizes 300 or 500. For the conditions with different intercepts, constrained models show excessively high biases for the slopes with unbalanced class proportion while the performance is found to be poor in most settings.

Though the results for two-predictor models are similar to single-predictor models, simulation design factors more systematically impact the results. The results for free estimating models can be summarized as follows. Compared to the single-predictor models, biases are lower in most conditions. The biases for large- and non-difference conditions are lower than the moderate ones in general. The inclusion of an intercept difference in the model improves the estimation results. However, constrained models show higher biases than free models for every condition, and the number of parameters with serious biases increases as the magnitude of difference in residual variances increases. In addition, with the largest sample size (SS = 2,000) or with a large disparity between residual variances under balanced class proportion, free models have low biases for most parameters. Under unbalanced class proportion settings, some estimates in large- and moderate-difference conditions show excessively biased values in residual variance of Class 1 even though the residual variance is correctly specified. Slope factors for Class 1 under moderate-difference conditions have low biases while some biases for the intercepts, residual variances, and the slopes of Class 2 are problematic. For the non-difference condition, biases are low as most of the parameters show the absolute values of bias under 0.1, but biases exceed 0.1 under sample sizes of 1,000 to a slight degree. However, when the residual variances are constrained to be equal between two classes, parameters are likely to be severely biased regardless of sample size and class proportion. The models with an intercept difference have excessive biases. To be specific, the parameter biases for large- and moderate-difference conditions with unbalanced class proportion show the worst results: all parameters exceed the acceptable bias level and significant biases of which the magnitude was over 1 were found. Slopes of Class 2 are biased downward in many conditions.

### MSE

**FIGURE 4:**
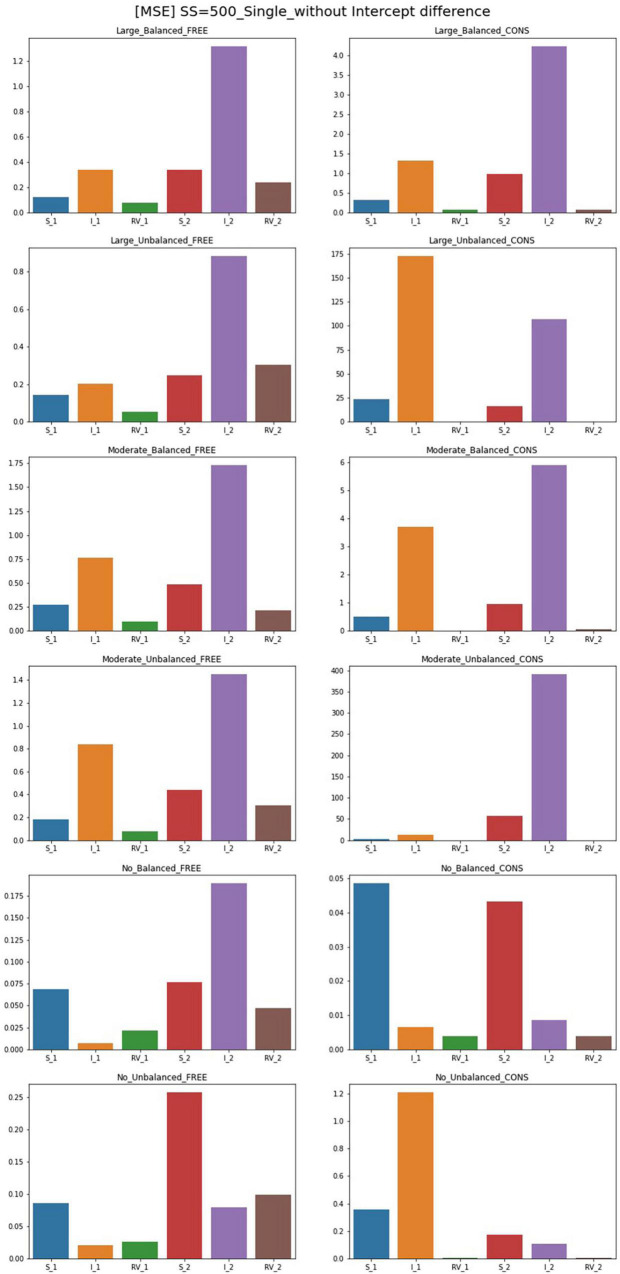
MSE of single-predictor models (sample size = 500, without intercept difference). Free, residual variances freely estimated; Cons, residual variances equally constrained; S_1, slope for class 1; RV_1, residual variance for class 1; S_2, slope for class 2; RV_2, residual variance for class 2.

**FIGURE 5:**
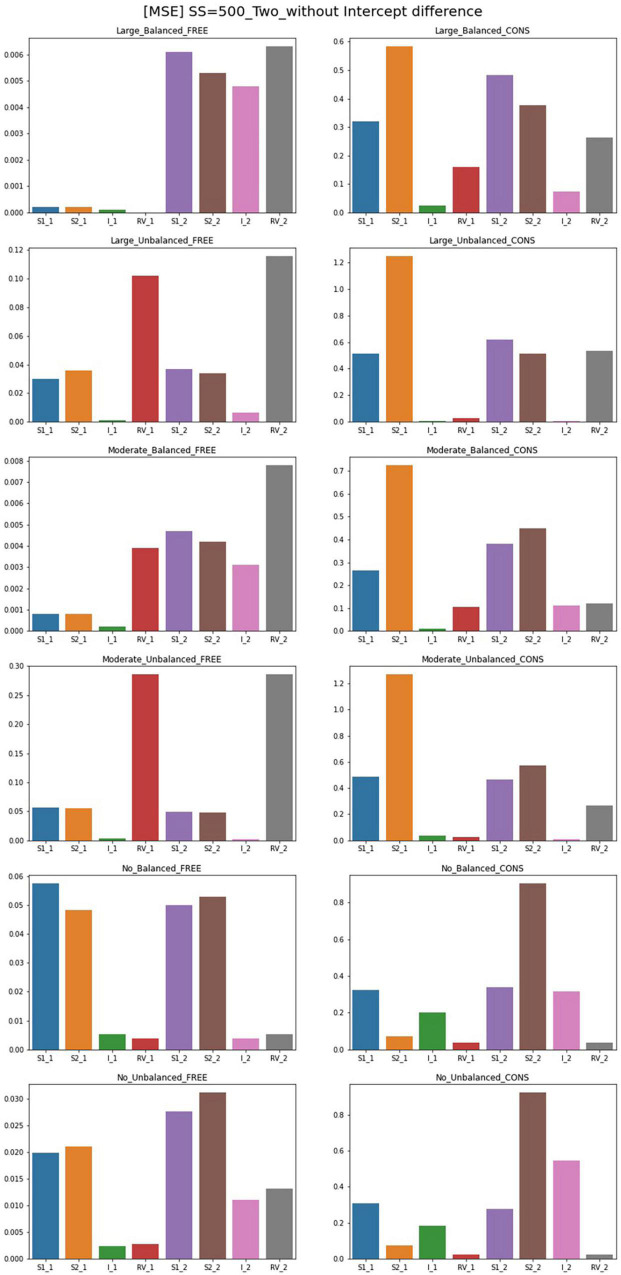
MSE of two-predictor models (sample size = 500, without intercept difference). Free, residual variances freely estimated; Cons, residual variances equally constrained; S_1, slope for class 1; RV_1, residual variance for class 1; S_2, slope for class 2; RV_2, residual variance for class 2.

The results for MSE for conditions of sample size 500 and intercept 0 are plotted in Figures 4, 5. For the single-predictor models in which the residual variances are freely estimated, MSE values are smaller regardless of class proportion when the sample size is large. However, parameters for the moderate-difference condition had high MSE in intercepts, which are even freely estimated. Constrained models show significantly poorer performance regarding MSE values when compared to the free models. When residual variances are constrained even though there is a big difference, the intercepts showed high MSEs than that of the slopes. Compared to other conditions, constrained models with sample sizes of 300 and 500 have larger MSEs for many parameters. When the sample size is 1,000, slopes and intercepts show high MSEs when those of residual variances are small. When the magnitude of the disparity between residual variances is moderate, the MSE of the constrained model is higher than the free estimation model but to a lesser extent than the large-difference condition.

When the population values of residual variances for each class are equal, freely estimated models show lower MSE values than other conditions; parameters of unbalanced class proportion show slightly higher MSEs than those of balanced conditions. Constrained models in these conditions also show undesirable results in slopes and intercepts when the sample size is 300. A sample size over 300 shows low MSE values. When an intercept difference is included in single-predictor models, the MSE values for free models generally decrease. The MSE values under a small sample size are also low whereas those of models without a difference between the intercepts are quite large. Under sample sizes of 300 and 500, MSEs are slightly larger; for balanced class proportion, sample sizes from 500 and for the unbalanced, values under the sample size of 1,000 are satisfactory.

When the residual variances are constrained, all the MSE values of the large-difference condition are substantially high, and those of the moderate-difference condition are also higher, but the value diminishes as the sample size increases. For the non-difference condition, although most of the values for other parameters only show a small degree of difference compared to the free model, that of the intercept in Class 1 is excessively high. When class proportion is unbalanced, the results of other parameters in the model become more notable that slope for both classes and intercepts for Class 2 become excessively high, which indicates it can result in problematic outcomes when the residual variances are constrained in these conditions although they have the same population value.

For the freely estimated multivariate models with an intercept difference of 1, except for some of the intercepts under unbalanced proportion conditions, most of the parameters have low MSEs. However, the constrained model shows higher MSEs; for the residual variance discrepant conditions, the parameters for Class 2 are poorly estimated. However, when the residual variances are the same, most of the parameters have lower MSE values except for the intercept in Class 1. Some MSEs for slopes are problematic but the degrees are not systematically impacted by the magnitude of the sample sizes.

### Coverage

**FIGURE 6:**
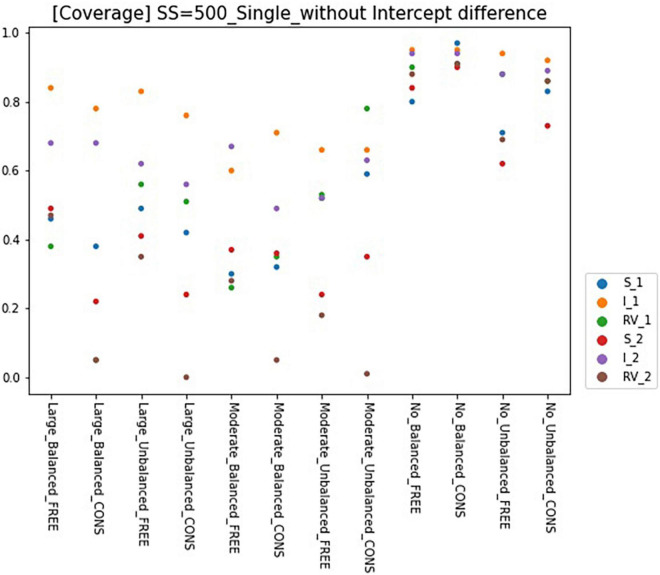
Coverage of 95% confidence interval of single-predictor models (sample size = 500, without intercept difference). Free, residual variances freely estimated; Cons, residual variances equally constrained; SS, sample size; CP, class proportion; RV, the disparity in the magnitude of class-specific residual variances; C1_S, slope for class 1; C1_I, intercept for class 1; C1_RV, residual variance for class 1; C2_S, slope for class 2; C2_I, intercept for class 2; C2_RV, residual variance for class 2.

**FIGURE 7:**
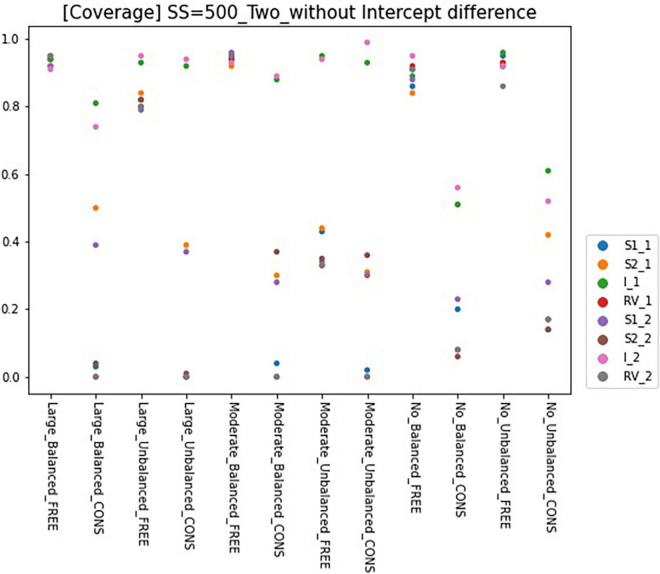
Coverage of 95% confidence interval of two-predictor models (sample size = 500, without intercept difference). Free, residual variances freely estimated; Cons, residual variances equally constrained; SS, sample size; CP, class proportion; RV, the disparity in the magnitude of class-specific residual variances; C1_S1, slope for x1 in class 1; C1_S2, slope for x2 in class 1; C1_I, intercept in class 1; C1_RV, residual variance for class 1; C1_S1, slope for x1 in class 2; C1_S2, slope for x2 in class 2; C2_I, intercept in class 2; C2_RV, residual variance for class 2.

Results for coverage of the 95% confidence interval for conditions of sample size 500 and intercept 0 are presented in Figures 6, 7 and can be summarized as follows. The coverage rates for the models with one predictor are below 0.9 for many conditions and thus, are unsatisfactory. When the intercepts are the same between the classes, some of the parameters show satisfactory coverage rates when the sample size is 500 and over under balanced class proportion conditions, and a sample size of 1,000 and over under unbalanced class proportion conditions. With large sample sizes (SS = 1,000 and 2,000), some conditions of large-difference and moderate-difference show quite fair coverage levels (but most of them were under 0.9, which could be problematic), considering that RMMs show a lower coverage rate in general. On the other hand, constrained models show considerable coverage rates under all large- and moderate-difference conditions. Parameters for non-difference conditions show a similar coverage rate to the free model and even show slightly higher rates than the free model. The intercept difference between the classes is present and also coverage rates for most of the parameters are inappropriate, compared to the desired level. Among those, a model with large-difference conditions in residual variances show fair coverage when the sample size is large (SS = 1,000 and 2,000) under either balanced or unbalanced class proportions. However, in the moderate- and non-difference, only models under unbalanced class proportions show a decent rate of coverage when the sample size is 1,000 or 2,000. When the constraints are imposed on residual variances, the coverage for the non-difference condition is lower than the model without an intercept difference.

When the model has two predictors, coverages for the free model are higher than the single-predictor model. Regardless of the intercept difference, coverages for most of the free models with balanced class proportion are satisfactory. The unbalanced model shows lower bias than the balanced model, except for the non-difference conditions. The coverage rates for the constrained model are seriously low except for one or two parameters in a model. Unlike the single-predictor models, coverages for all non-difference conditions are low, even though the residual variances are equal.

To summarize the results, when a sole predictor is included in the model, even with a correctly specified model, bias and coverage can be poor. When conditions of balanced class proportion, a sample size over 1,000, and large class separation are secured, better estimates can be obtained in a single-predictor model. When two predictors are included in the model, correctly specified RMMs can result in better estimates than a single-predictor model. When class-specific intercepts are different, results for all criteria are satisfactory under most of the conditions. However, even in a two-predictor model, for some conditions, problematic outcomes can result: small sample sizes, unbalanced class proportion, and low class separation. Even though there are some conditions with accurate estimates even below the minimum suggested sample size of 3,000 in correctly specified models, models with equality constraints on residual variances yield inaccurate estimates under most conditions when the sizes of residual variance are discrepant between the two classes. Under these discrepant conditions, most of the parameter estimation results are inaccurate. Especially when the discrepancy between the class-specific residual variances is large, the problematic results for bias and coverage are severe. Under the conditions that the residual variances are actually the same in the population value, the results are problematic except for some conditions; the single-predictor models without an intercept difference with a sample size over 500 are well estimated, and better estimates are obtained than the free model. Although the free models under these conditions result in poor estimates when the sample size is small especially in coverage, restricted models show satisfactory estimates considering all criteria under these conditions. The impacts of constraints are more remarkable when the two predictors are included, and the class-specific intercepts are different, which show more excessively biased results than the single-predictor models with inappropriate constraints.

## Real Data Application

### Regression Mixture Analysis to Explore the Differential Effects of Smartphone Dependency and Depression Among Adolescents

In this section, we will provide an example of analyzing regression mixtures with and without equality constraints on residual variances with a small sample size. This applied example will provide a simple and practical guide on modeling with RMMs, focusing on situations where researchers can confront problems with estimation and may seek for methods (such as constraining the residual variances) to solve it.

We used the 5th wave of the panel data collected from the “Research on Juvenile Panel for Aborting Education” by the Korea Youth Policy Institute. This data set includes several variables concerning adolescents who dropped out of school including their relationships with parents, teachers, and friends; daily patterns of spending time; and psychological traits. The sample included 318 adolescents who dropped out of middle or high school. This sample size may be considered insufficient for regression mixtures. In general, RMMs require a large sample (i.e., more than 3,000) for a stable analysis. As our simulation study is focused on circumstances where the dataset is unideal, such as with small samples, we chose a situation where the sample is less than 500 in order to demonstrate the results from the simulation study.

The study explored whether the differential effects between smartphone dependency and depression exist among adolescents. We were motivated by the literature of Zhao and Lapierre (2020) which implied that the impact of problematic smartphone use on an individual’s stress and depression can differ among individuals, for example being affected by a given individual’s purpose of smartphone use or level of social support. Starting from this literature, we aim to figure out whether there are hidden subgroups indicating the effect (both in direction and magnitude) of smartphone dependency on depression. The single-predictor models where the intercept and slope were allowed to vary across the classes have been used in order to intuitively compare these results with the simulation results. For the free models, residual variances were also allowed to be variant between the classes; they were constrained to be equal across classes in restricted models.

The fit statistics of each enumeration phase are given in Table 7. We compared the fit indices by increasing the number of latent classes from 1 to 3. As it is known that AIC (Akaike Information Criterion) often over extracts the latent classes in regression mixtures, we used BIC Bayesian Information Criterion) (for selecting the number of the classes. For both free and restricted models, two-class models were selected.

**TABLE 7:** Fit indices for free and restricted models of data-application example.

	1-class	2-class	3-class
**Free model**			
Loglikelihood	−196.065	−183.02	−175.588
Parameters	3	7	11
AIC	398.129	380.04	373.176
BIC	409.415	406.375	414.559
ABIC	399.9	384.172	379.669
Entropy	–	0.343	0.676
Class proportions		–	–
Class 1	1	0.405	0.314
Class 2	–	0.595	0.582
Class 3	–	–	0.104
**Restricted model**			
Loglikelihood	−196.065	−183.965	−176.2
Parameters	3	6	9
AIC	398.129	379.929	370.4
BIC	409.415	402.502	404.258
ABIC	399.99	383.471	375.712
Entropy	–	0.757	0.667
Class proportions	–	–	–
Class 1	1	0.844	0.412
Class 2	–	0.156	0.107
Class 3	–	–	0.481

Next, we estimated the two-class models with and without the equality constraints on residual variances. For the two-class models, the restricted model showed an improved entropy value (0.343 in free model, 0.757 in the restricted model). In Table 8, the results of the estimation, including the parameter estimates, standard errors, and *p*-values, are provided. In the free model in which the residual variances are allowed to differ between the classes, the characteristics of each two groups can be distinguished with the magnitude of the slope. Individuals in Class 1 comprised 40.4% of the sample and showed a stronger indication of the effect of smartphone dependency on depression compared to those in Class 2. The intercepts of these classes were similar in magnitude (difference = 0.14). However, when the residual variances are constrained (even though the class-specific residual variances estimated in the free model are not the same), the estimation results were clearly different from the results of the free model. While the class proportions of free models were more balanced (40.4: 59.5), in the restricted model, the class proportions were significantly unbalanced (84.4: 15.6). The directions of the effects were the same (+) but they differed in magnitude. In the restricted model, it is possible that the differential effects were not effectively captured, as the slopes of each class are very similar in magnitude and the difference is 0.057.

**TABLE 8:** Parameter estimates, standard errors for 2-class models of free and restricted models of data-application example.

	Class 1			Class 2		
	*B*	*SE*	*p*	*B*	*SE*	*p*
**Free**						
Intercept	1.27	0.24	0.00	1.13	0.09	0.00
Slope	0.36	0.09	0.00	0.16	0.07	0.02
Residual variance	0.19	0.02	0.00	0.09	0.02	0.00
**Restricted**						
Intercept	1.12	0.08	0.00	1.84	0.37	0.00
Slope	0.21	0.04	0.00	0.27	0.14	0.05
Residual variance	0.11	0.01	0.00	0.11	0.01	0.00

Although these results may not be straightforwardly helpful in comparisons of the performance of the free and restricted models since the true model derived from applied research cannot be known in advance, this real data application demonstrates how inappropriately imposing equality constraints can distort the estimation results, even though these constraints may lead to better indices regarding convergence. Therefore, we recommend not to constrain class-specific residual variances with small samples especially when the absolute value of effects (demonstrated by slope) is different between the classes.

## Conclusion and Discussion

This study conducted a Monte Carlo simulation study to examine the performance of freely estimated and restricted RMMs in parameter estimation and compared the results of the two models. Data were generated for the two-class model, and four types of models were considered: single and two-predictor models with two different settings for class-specific intercepts. Manipulated factors for simulation design are the magnitude of difference with class-specific residual variance, which are set by varying the class specifications, sample size, and class proportion. Under manipulated conditions, four models were estimated twice, one with freely estimating residual variances and one with restricted residual variances. To evaluate parameter estimation accuracy, the criteria of parameter estimate bias, MSE, and coverage were considered comprehensively.

The results can be summarized as follows. For the free estimating models, although the parameter estimates were fairly obtained, only a few of them met the criteria, and bias and coverage were especially problematic under many conditions. But under the following conditions, more accurate estimates were obtained: models with two predictors, large sample sizes (SS = 1,000 and SS = 2,000), a large difference in regression weights between the classes (0.7/0.2, 0.5/−0.5), and balanced class proportion. These results for free models are consistent with the study of Jaki et al. (2019) where coverage rates were slightly lower than the acceptable level even when the parameter estimates were close to the true value, parameter estimates were inaccurate for sample sizes of 500 and below, and lower accuracy was found in decreased class separation settings. For the constrained models, the estimates were unfavorable except for some cases. When the residual variances were unequal between the classes, estimates were very poor under all settings. A large discrepancy in residual variances between the classes led to worse results. Models with a smaller discrepancy for those were also problematic under most conditions, but to a lower degree.

Moreover, although good estimates were expected when the values for residual variance were equal between the classes, the results were also poor in many cases. These results were inconsistent with Kim et al. (2016) where the constrained models under the discrepant condition generally resulted in better estimates than the free model. It can be expected that even a slight model misspecification led to a problematic result in smaller sample size settings as previous studies have reported that RMMs are very sensitive to model assumptions and specification (Lamont et al., 2016; Wadsworth et al., 2018; Sherlock et al., 2021).

Furthermore, the patterns of problematic consequences that were different from the results of Kim et al. (2016) can be summarized as follows. First, the type of parameters with serious problems was different. Unlike the previous study, where only the slopes were biased, in this study, the intercepts and residual variances including the slopes were also biased. In particular, the intercepts were seriously biased in many cases in this study while intercepts were accurately estimated even in harsh conditions in Kim et al. (2016). Second, although their study resulted in bias for both classes, parameters in Class 2 resulted in more serious bias in this study. This result is similar to Jaki et al. (2019) in that the coverage rates for classes with smaller slopes were more severe. These different results may have been caused by the model instability of RMMs (Jaki et al., 2019), considering that the simulation conditions set in this study are not ideal.

Therefore, taken together with the results from Kim et al. (2016) and Jaki et al. (2019), the recommendations for researchers using RMMs are proposed as follows. First, for researchers using smaller sizes than the recommended minimum size of 3,000, the equality constraints on class-specific residual variances should not be imposed when the residual variances are discrepant. With the discrepant conditions, even problems in estimation were confronted when using data of small sample sizes, reducing the number of parameters can lead to more serious problems than the correctly specified models. Thus, in this case, it is always recommended to use a sample size of more than 500 and correctly specify the residual variance component. Second, considering the free models with sample sizes of 500 and below showed problematic results under most conditions, when it is unavoidable to use small samples, such circumstances are required for better estimation: more than one predictor, larger difference in slopes or intercepts between the classes, and balanced class proportion. Lastly, even in situations where residual variances are equal across classes, equality constraints should be imposed with caution when the sample size is under 3,000. Although restricted models mostly showed better performance than free models with large samples (Kim et al., 2016), when the sample size is small, only under conditions of “a single-predictor model with both class intercept zero and sample sizes of 500, 1,000, and 2,000,” was the accuracy of parameter estimates satisfactory, even better than that of free model. Thus, researchers should keep in mind that in some situations when residual variances are the same within a class, the constraints could lead to more accurate estimation than the free estimation model.

The contributions of this study are as follows. First, this study included diverse and realistic conditions that are prevalent in applied study settings in systematically examining the consequences of the correctly specified and the misspecified model and comparing the results of the two. Previous simulation studies are limited on this topic, and the existing study on RMMs only included ideal conditions, which is irrelevant to most applied researchers using data under realistic conditions. To reconcile this discrepancy between simulation research and applied research, this study included a small sample size starting from 300 and unbalanced class proportions that are often encountered in applied research settings. Specifically, researchers applying RMMs with less than ideal data could refer to the findings in the current study to use a model in a more appropriate way. Second, this study is meaningful in that it revealed the impact of residual variances in RMMs while the aspects of residual variance components have not been thoroughly examined in finite mixture modeling.

Despite the significance of this study as described above, some limitations exist that are recommended to be discussed in future researches. First, the conditions of this study are limited to specific circumstances. This study builds upon the conclusions of previous literature of Kim et al. (2016) by including small sample sizes and unbalanced class proportions, both of which are often encountered in applied research settings. Therefore, more factors can be manipulated in future research, such as the number of classes or interaction between the predictors in multivariate models. Next, this study is limited to a situation where the model is quite simple, as the covariates are not included in its scope. As these constraints could result in different results when the model is more complex in RMMs, it is recommended that future researchers conduct research with more complicated RMMs to thoroughly investigate methods of dealing with non-convergence issues by using equality constraints in RMMs.

## Data Availability Statement

The raw data supporting the conclusions of this article will be made available by the authors, without undue reservation.

## Author Contributions

JC designed the study, carried out the simulation, and wrote the manuscript. SH directed the research and aided in interpreting the results. Both authors contributed to the article and approved the submitted version.

## Conflict of Interest

The authors declare that the research was conducted in the absence of any commercial or financial relationships that could be construed as a potential conflict of interest.

## Publisher’s Note

All claims expressed in this article are solely those of the authors and do not necessarily represent those of their affiliated organizations, or those of the publisher, the editors and the reviewers. Any product that may be evaluated in this article, or claim that may be made by its manufacturer, is not guaranteed or endorsed by the publisher.
