# Supplementary material for: The Impact of Imposing Equality Constraints on Residual Variances Across Classes in Regression Mixture Models
Source: Front Psychol. 2022 Jan 27;12:736132. doi: 10.3389/fpsyg.2021.736132 (PMC8829145; doi:10.3389/fpsyg.2021.736132)

Supplementary Material

**Bias**

## Single-predictor model, without intercept difference


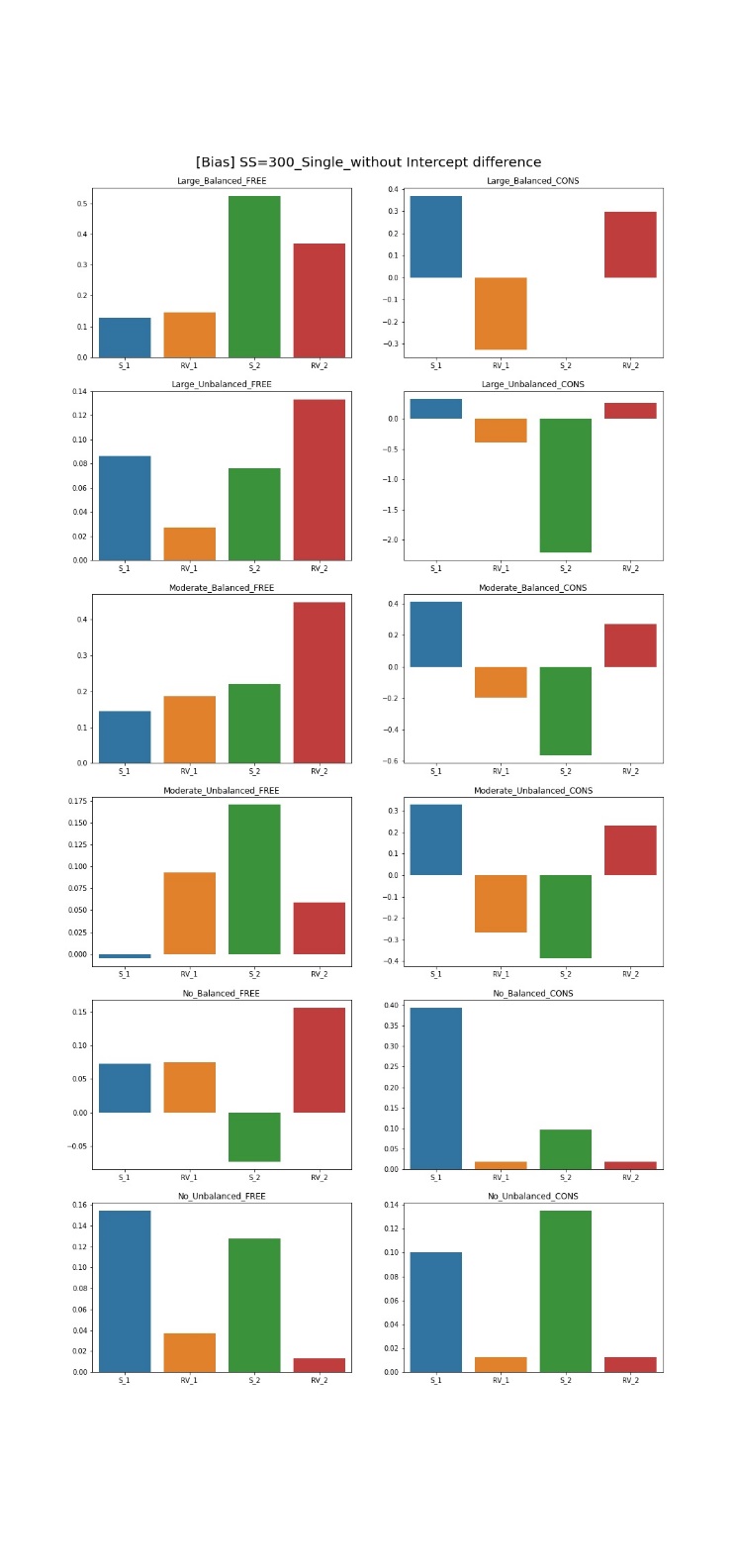


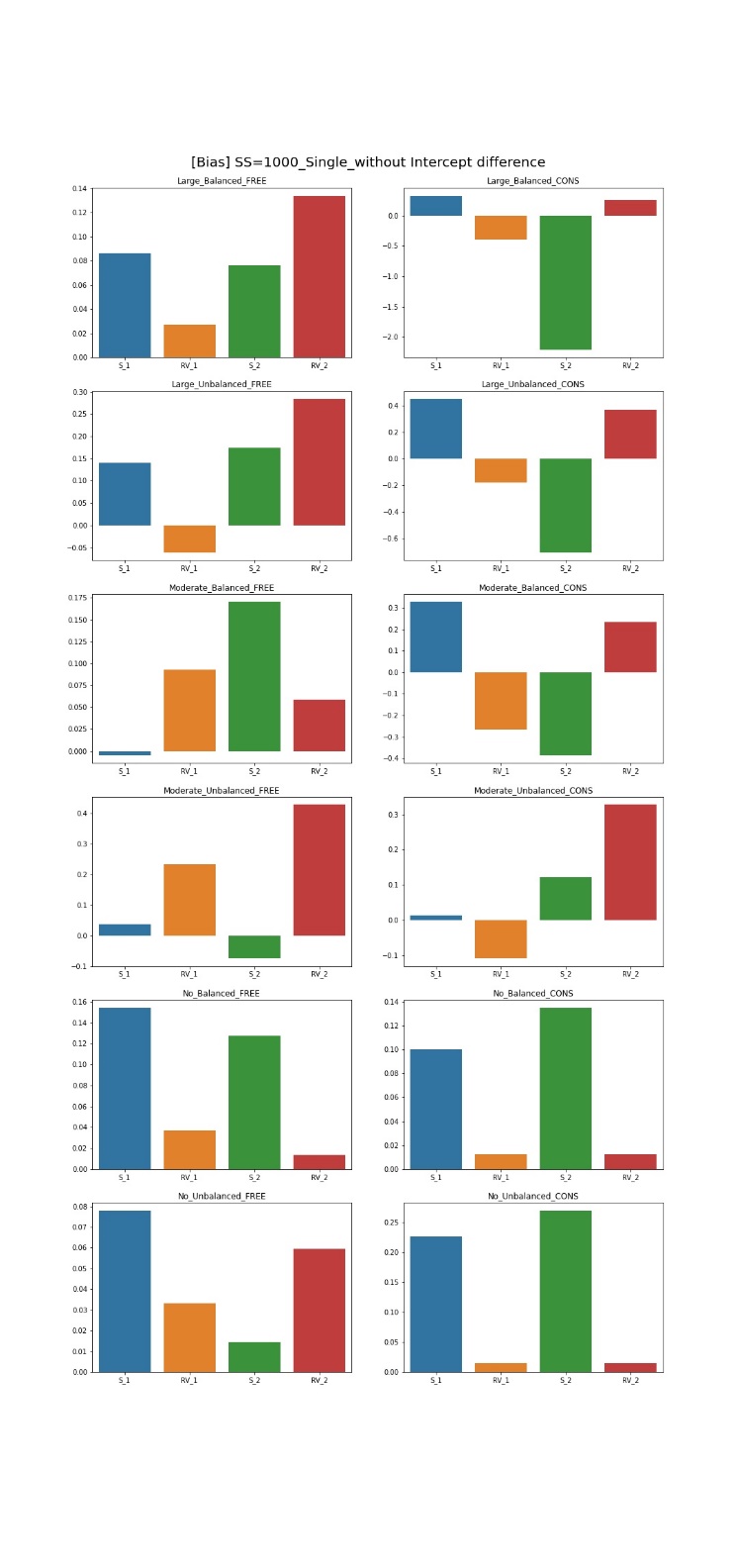

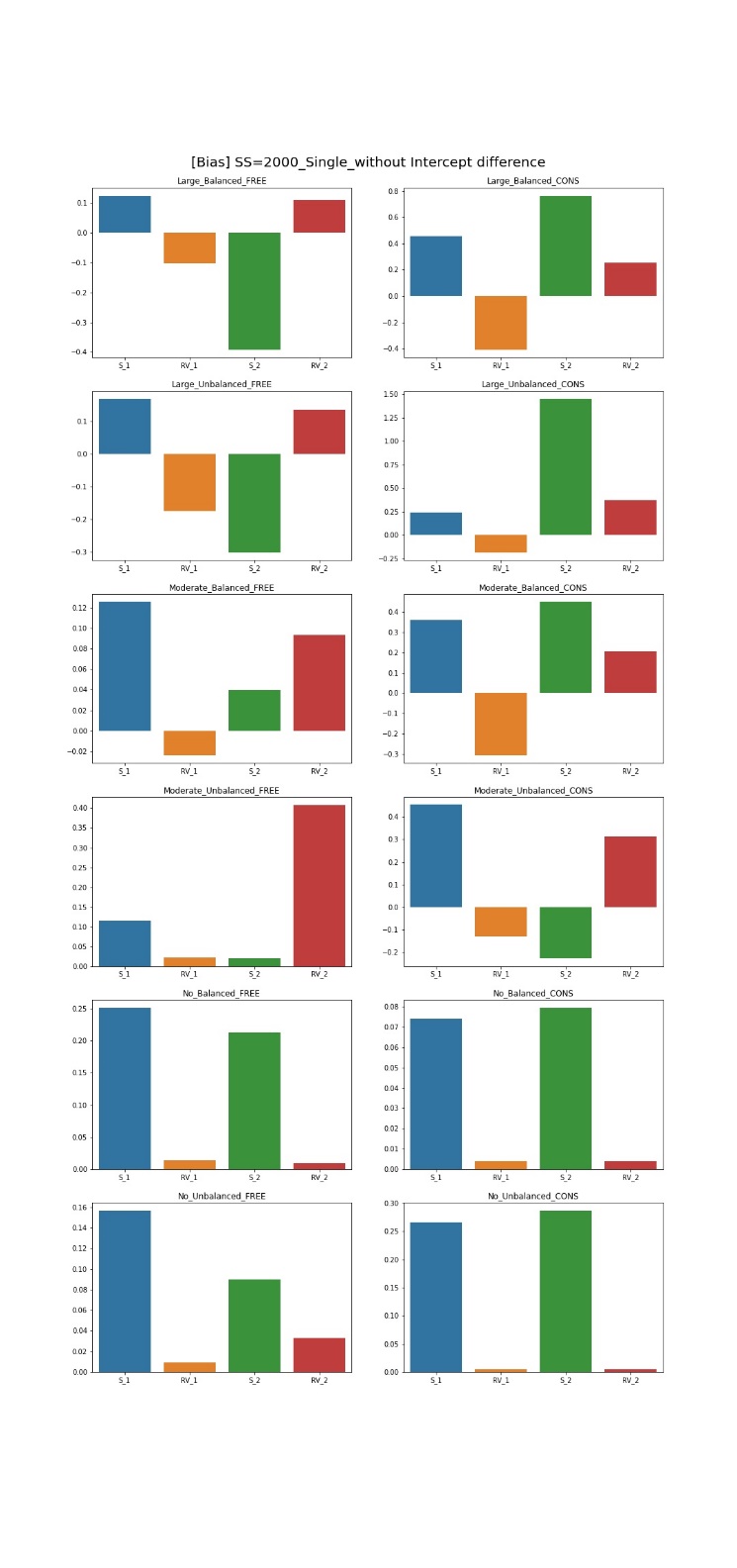


## Single-predictor model, with intercept difference


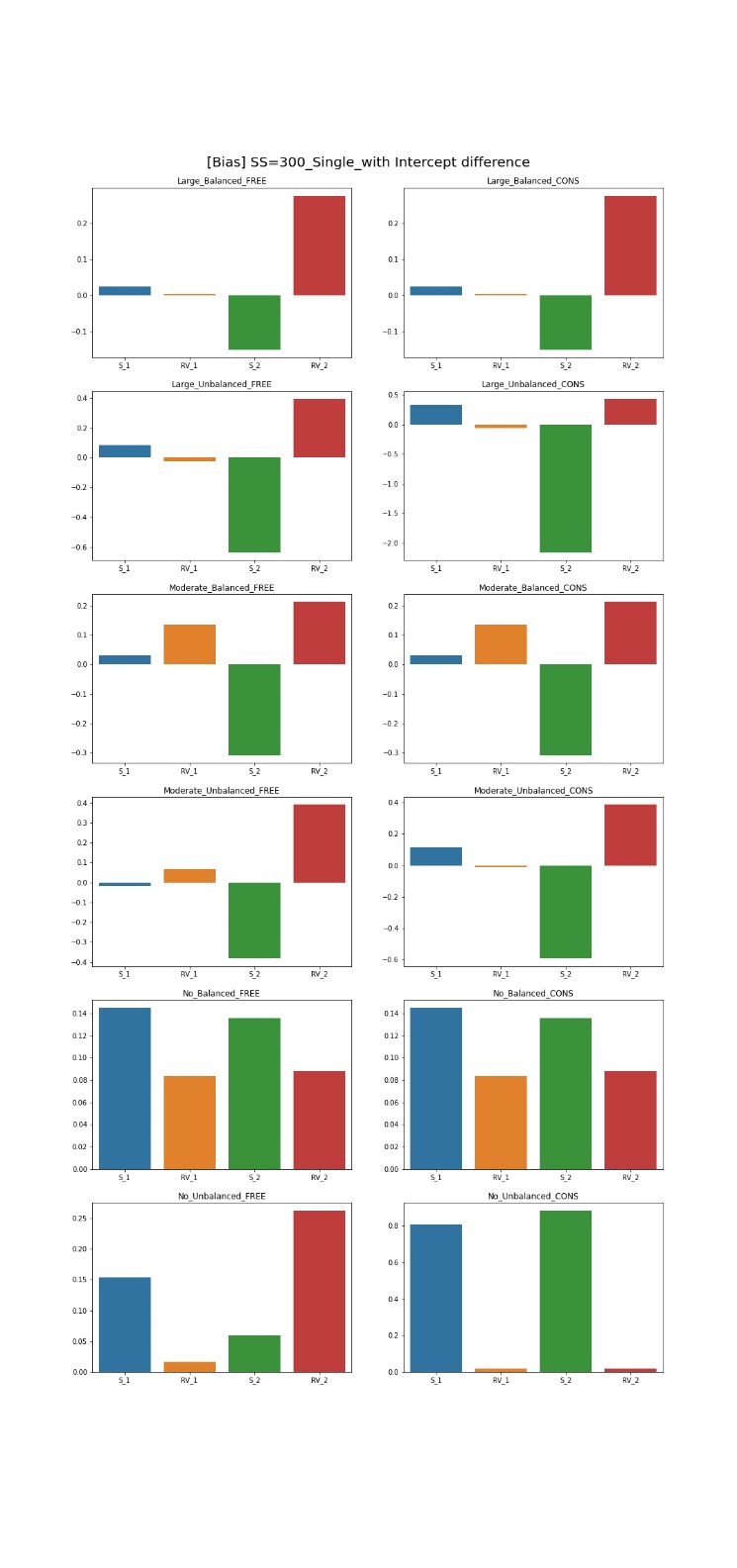

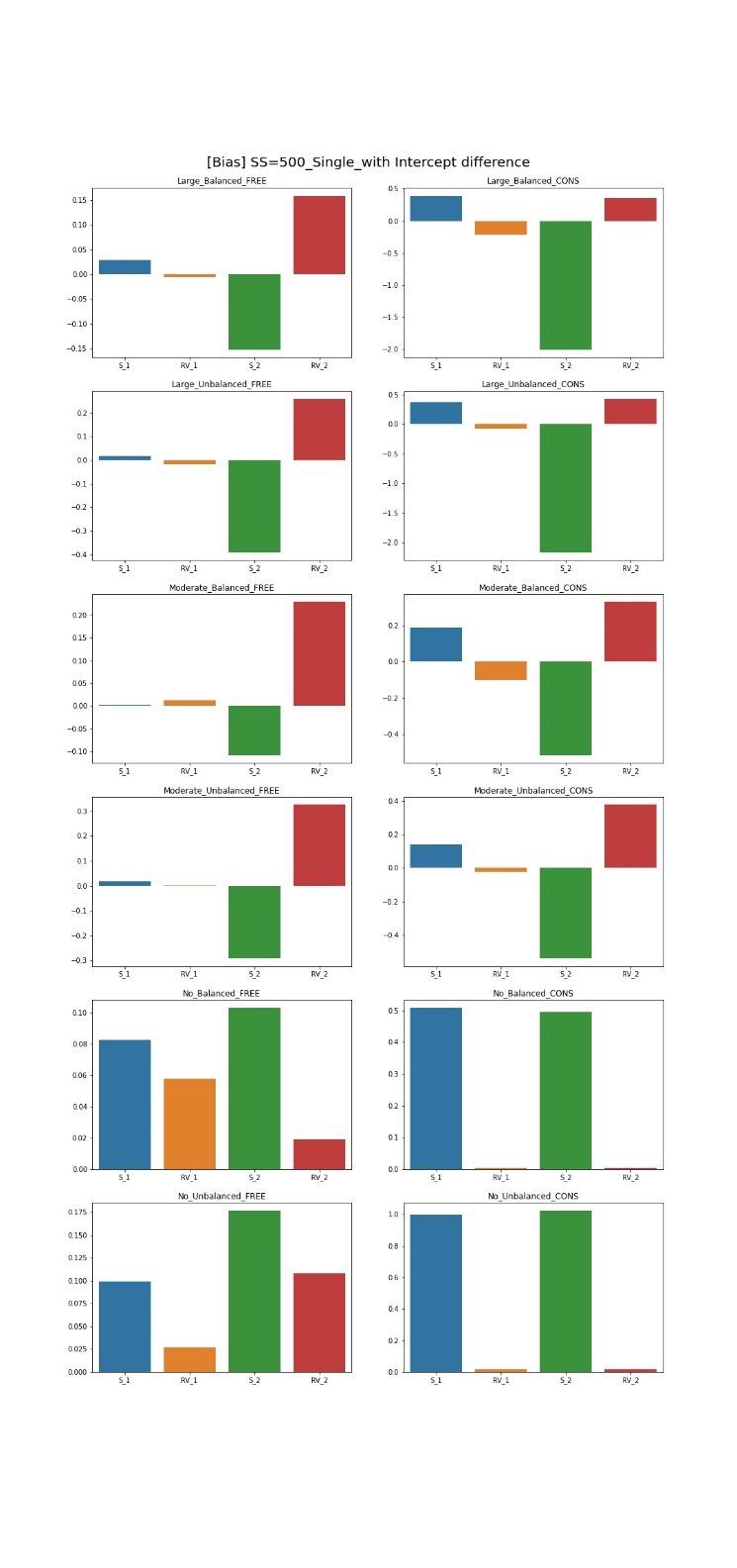


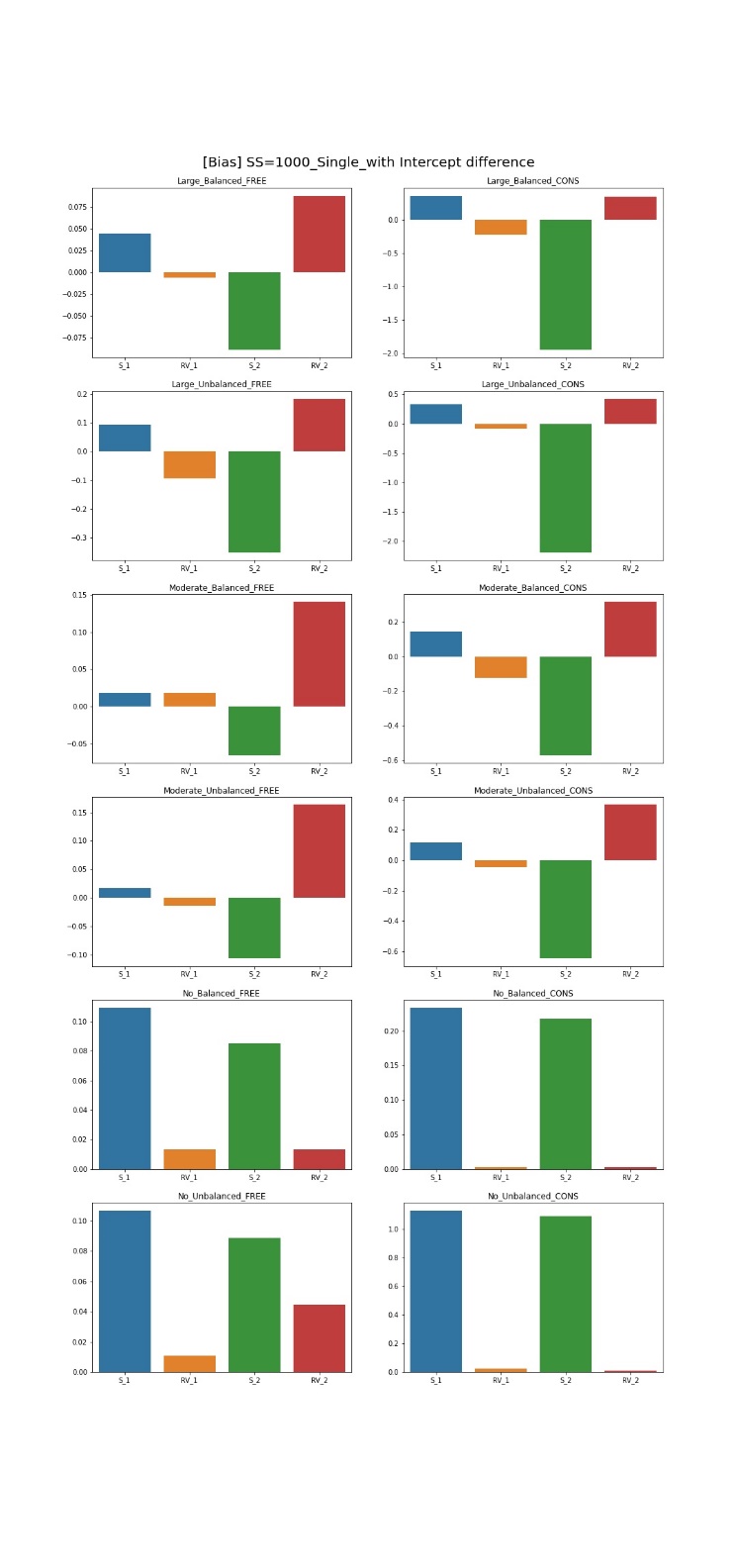

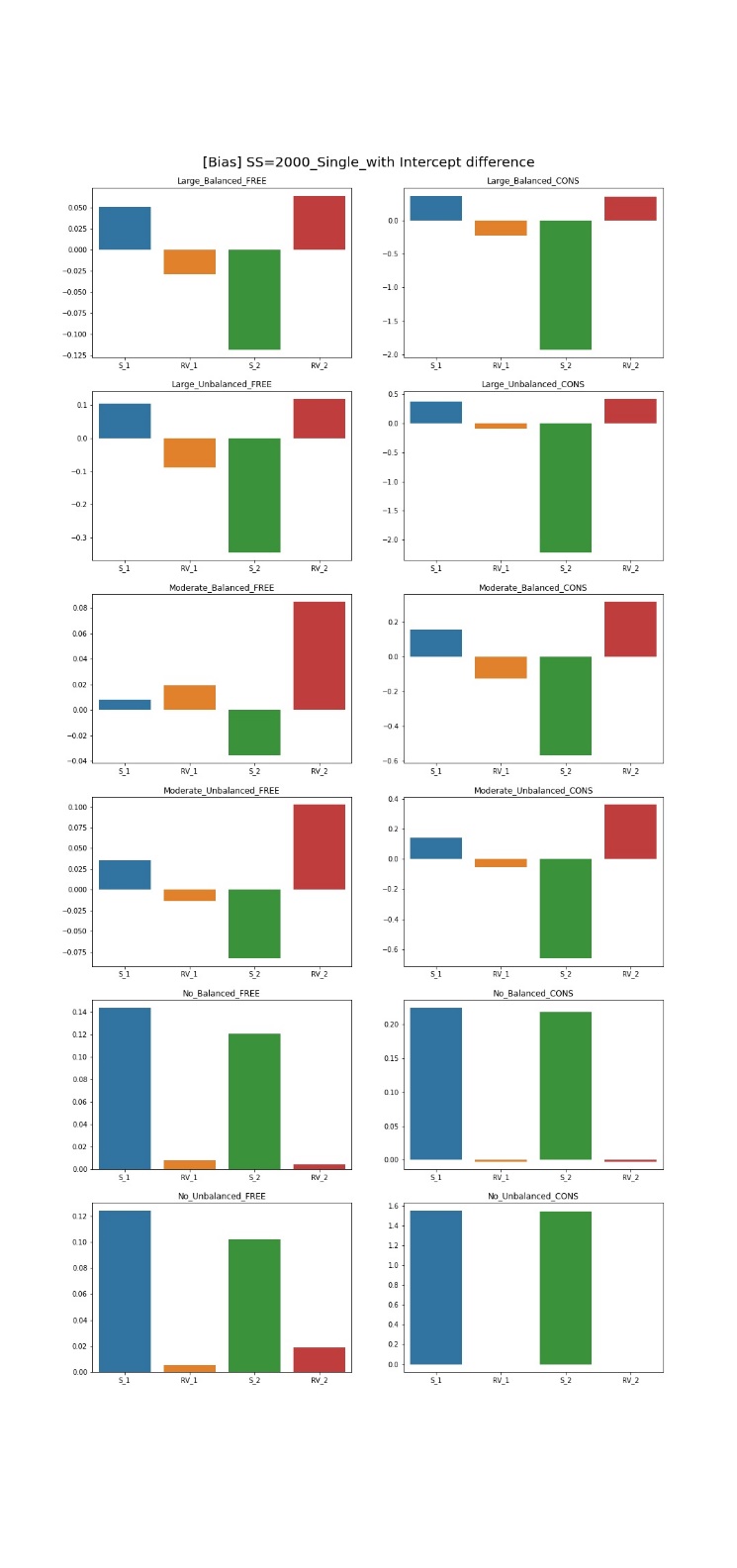


## Two-predictor model, without intercept difference


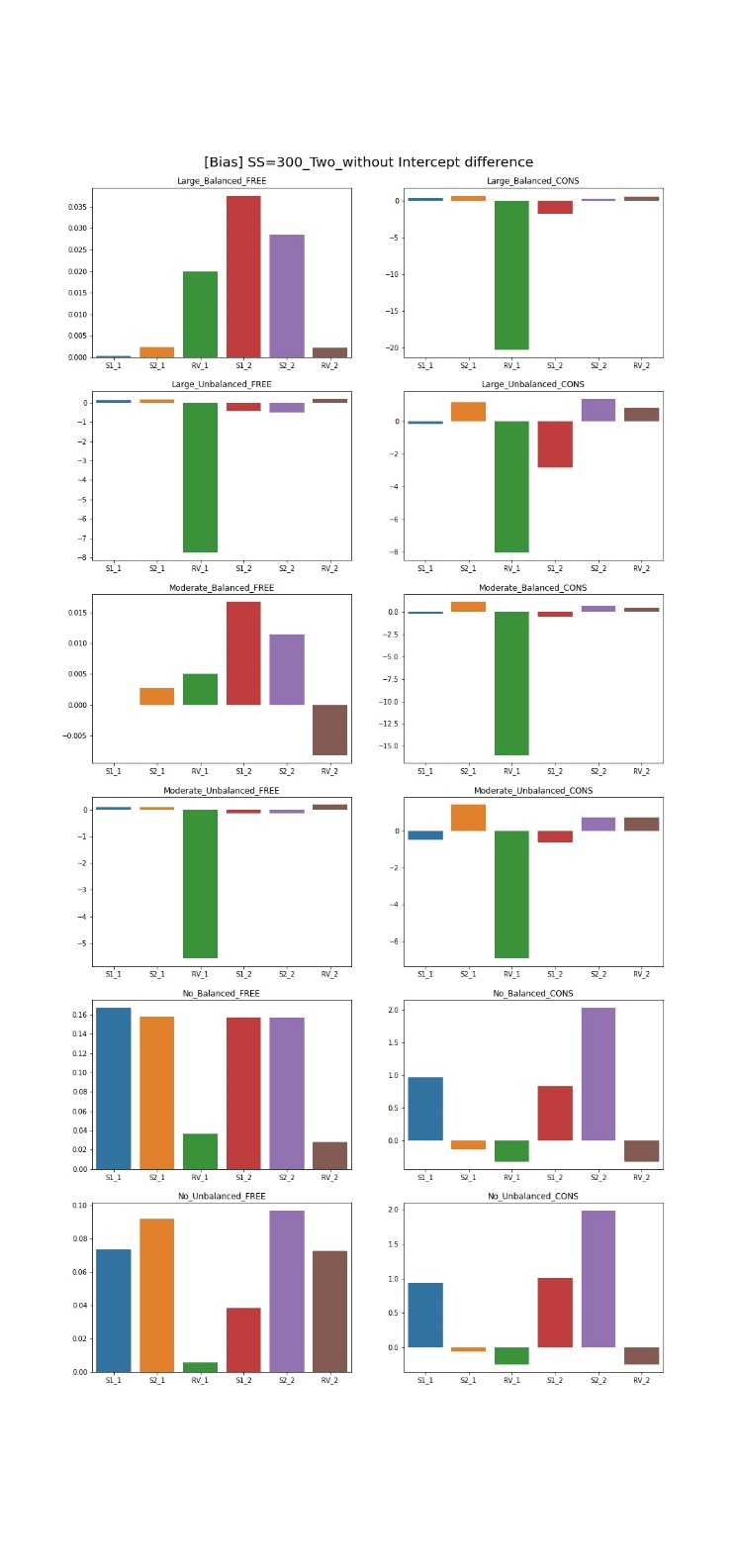


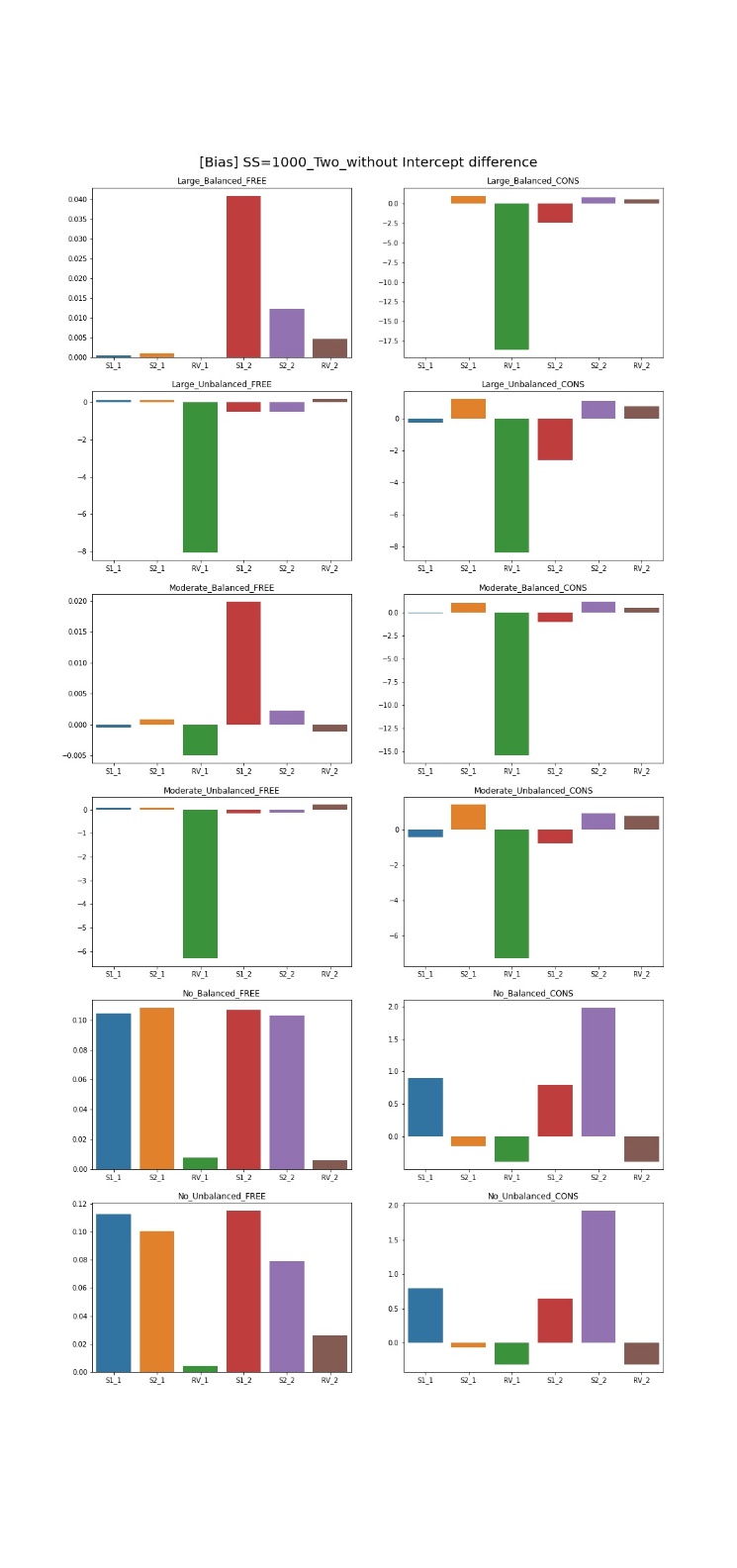

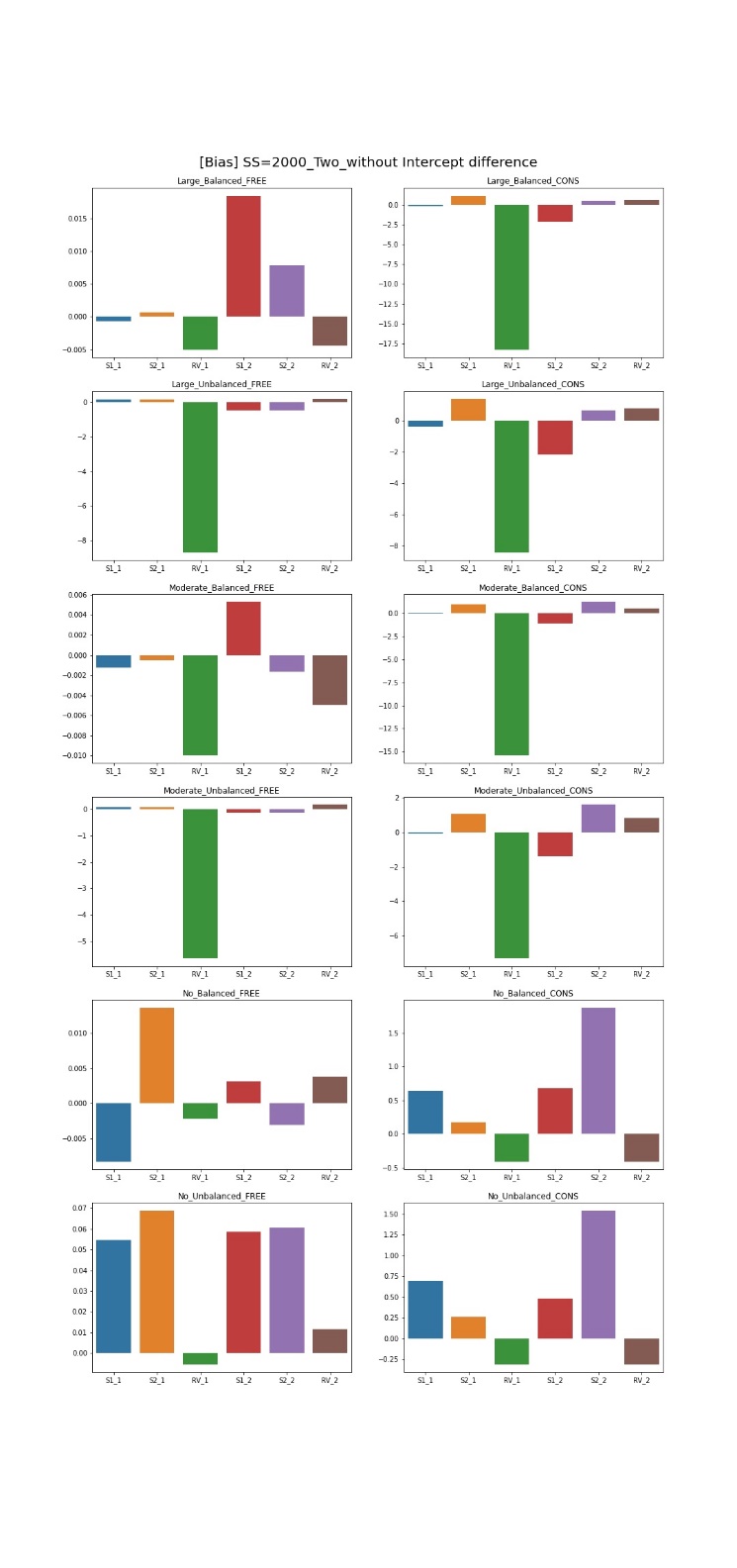


## Two-predictor model, with intercept difference


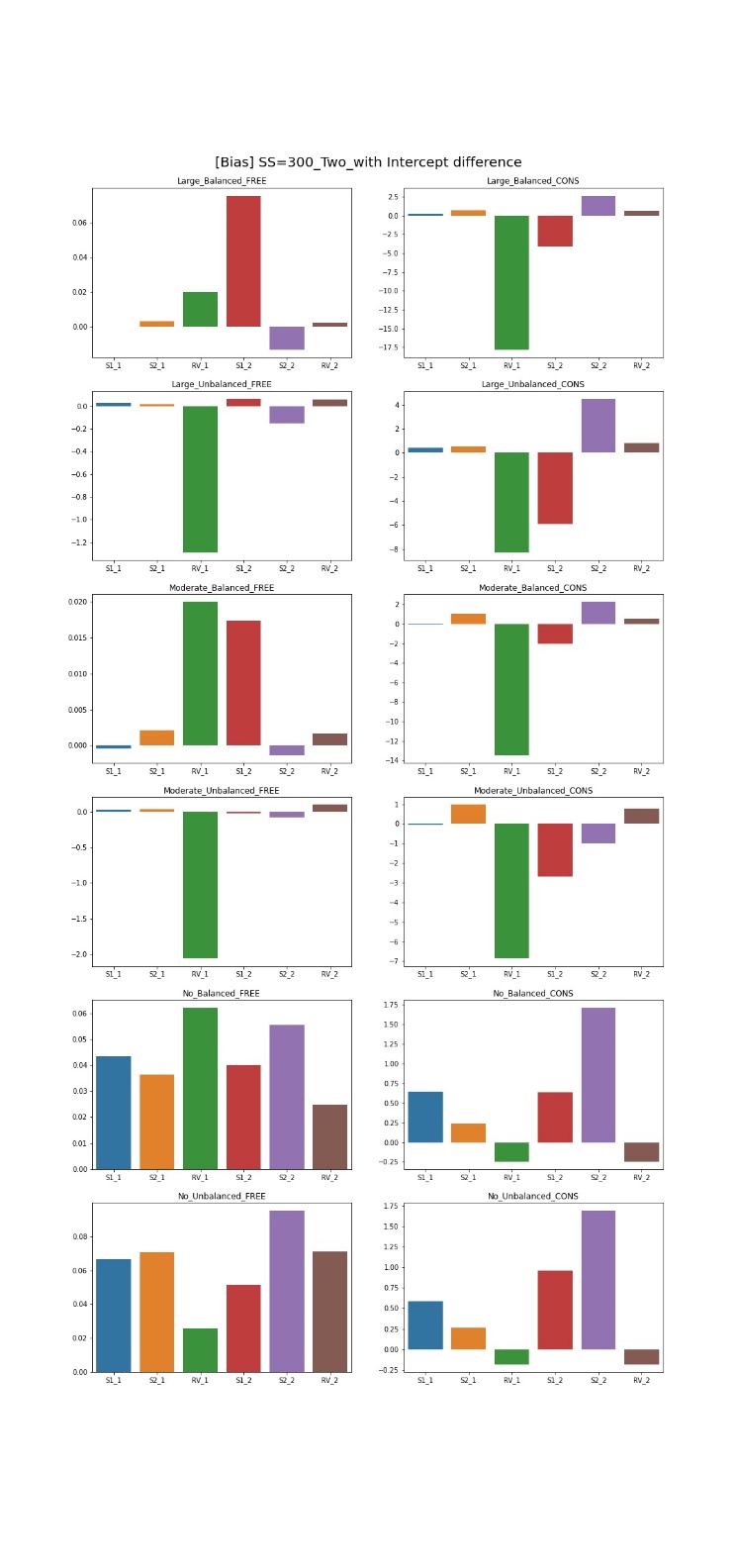

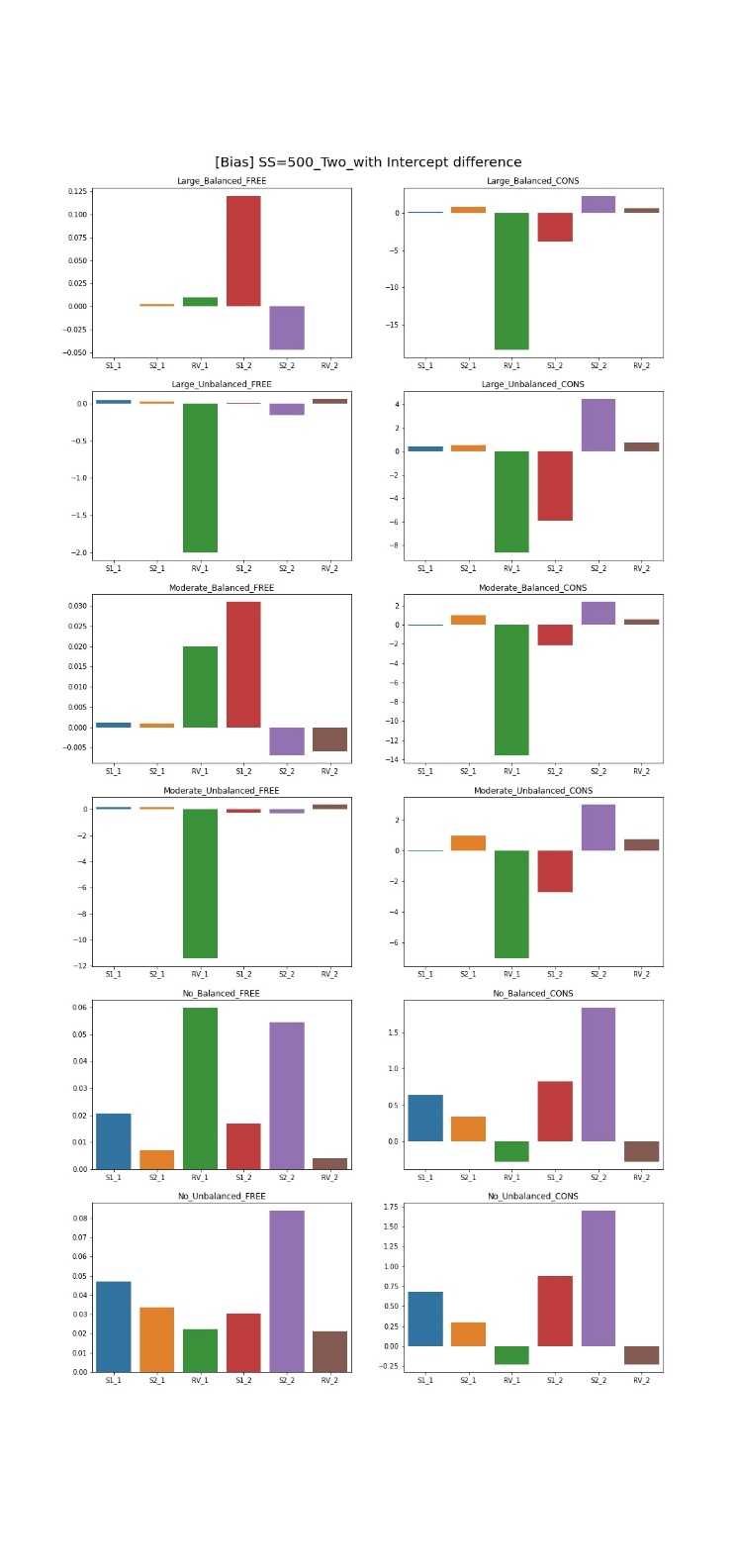


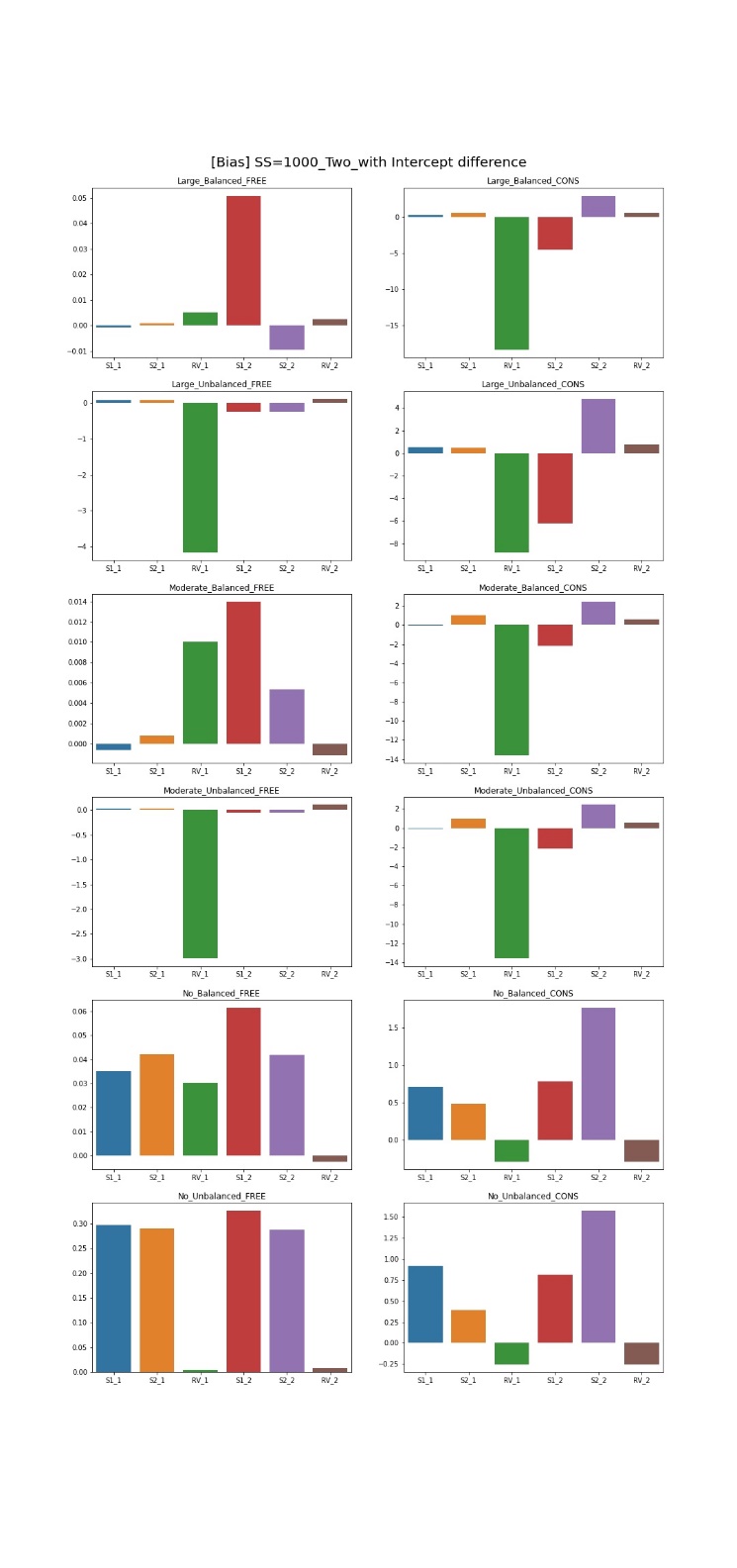

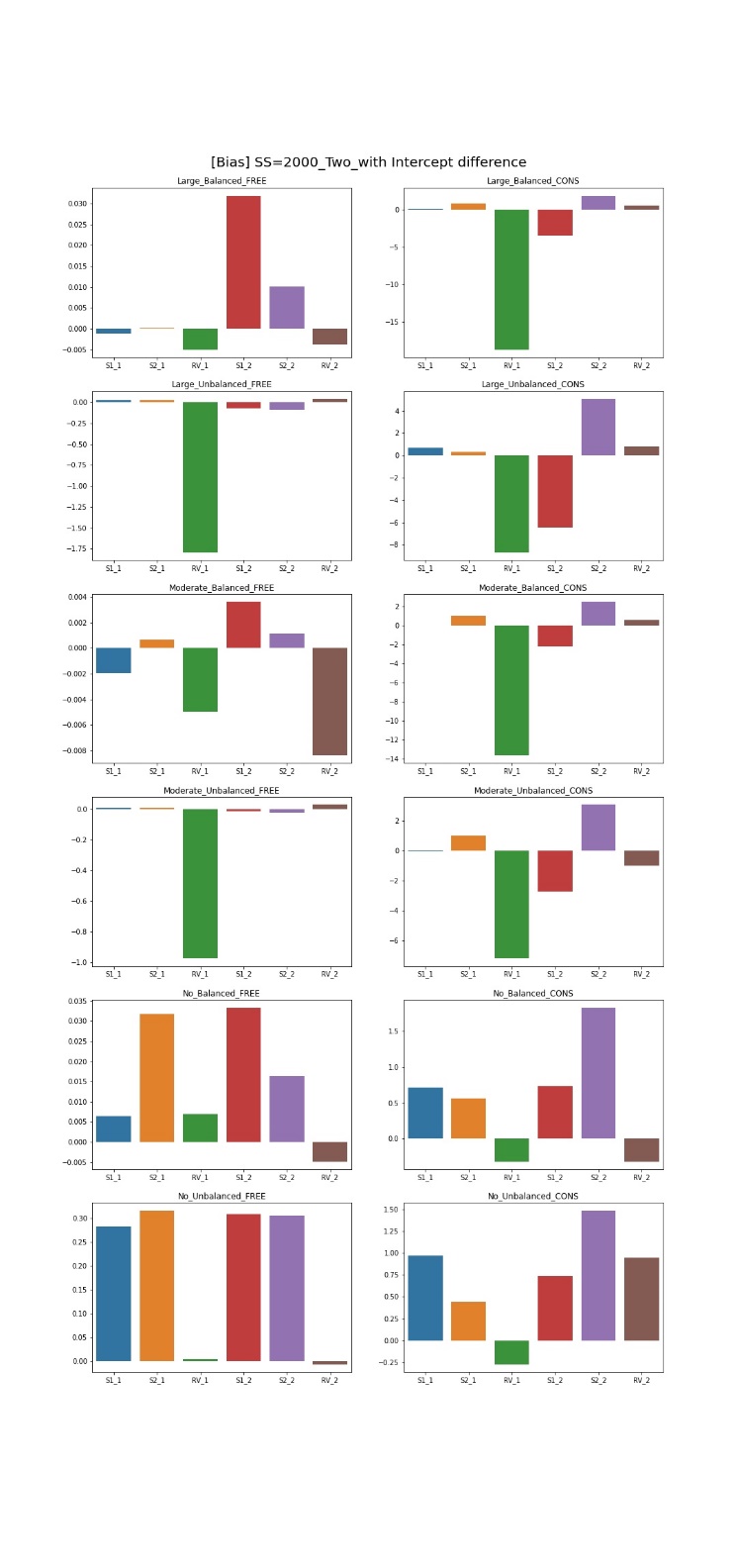


**MSE**

## 2.1 Single-predictor model, without intercept difference


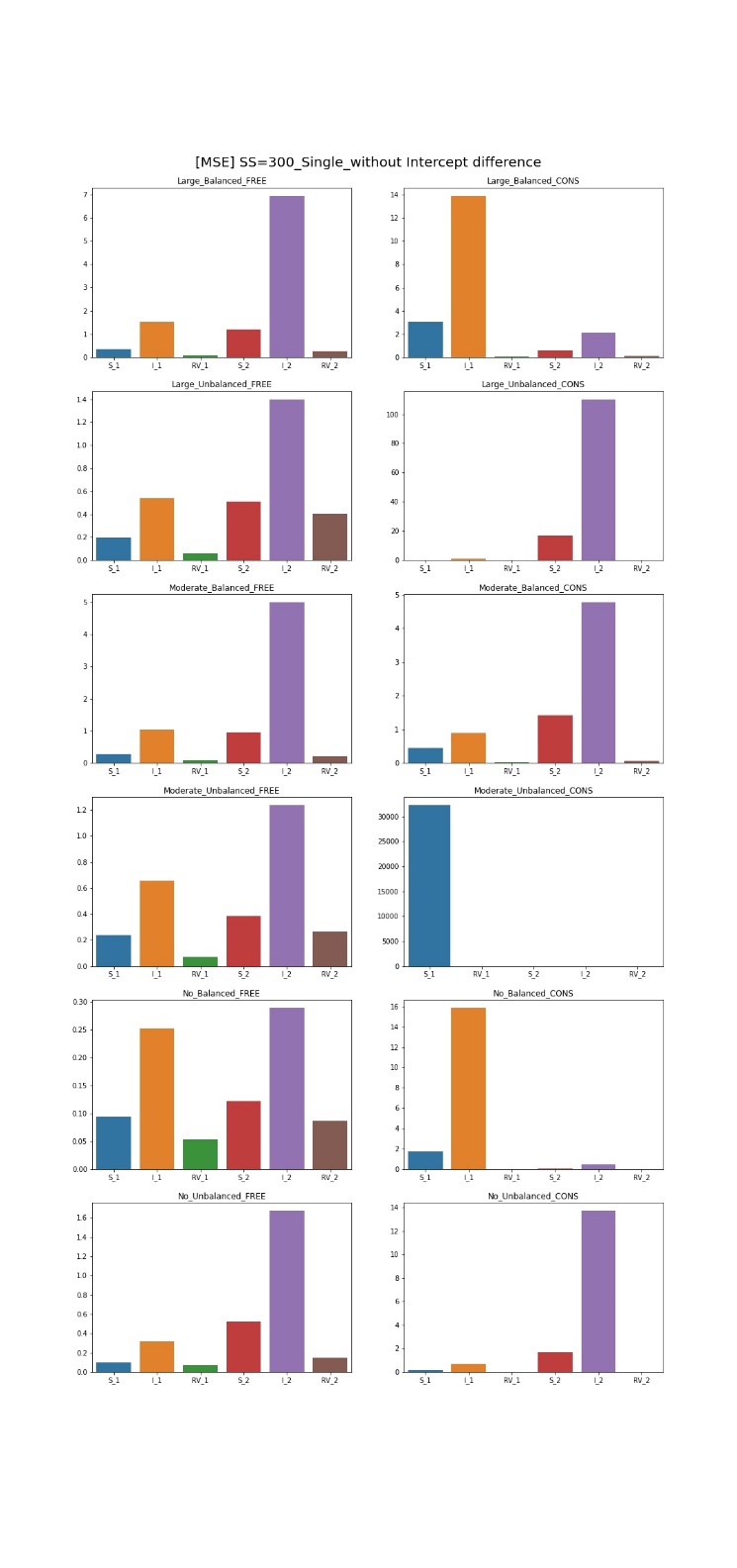


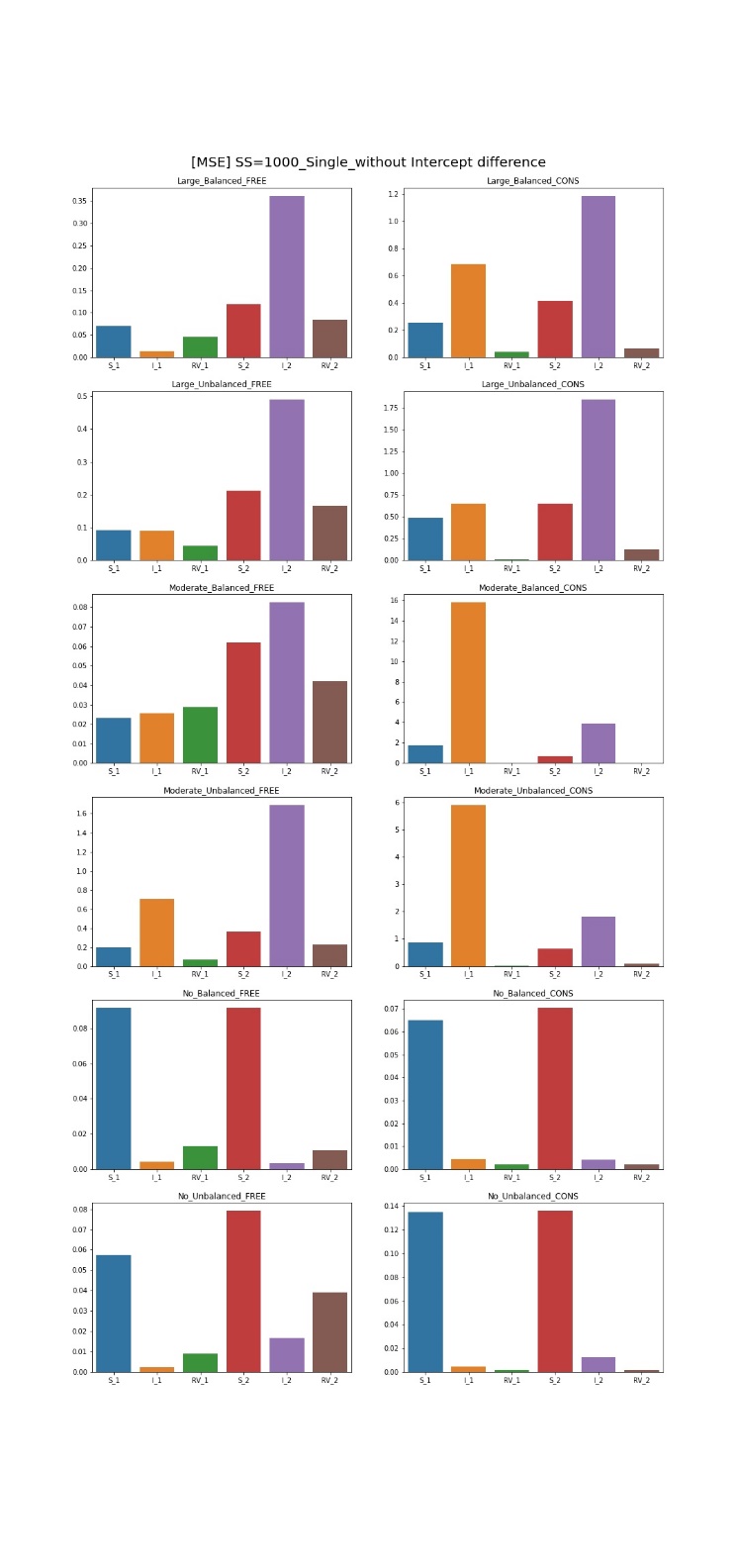

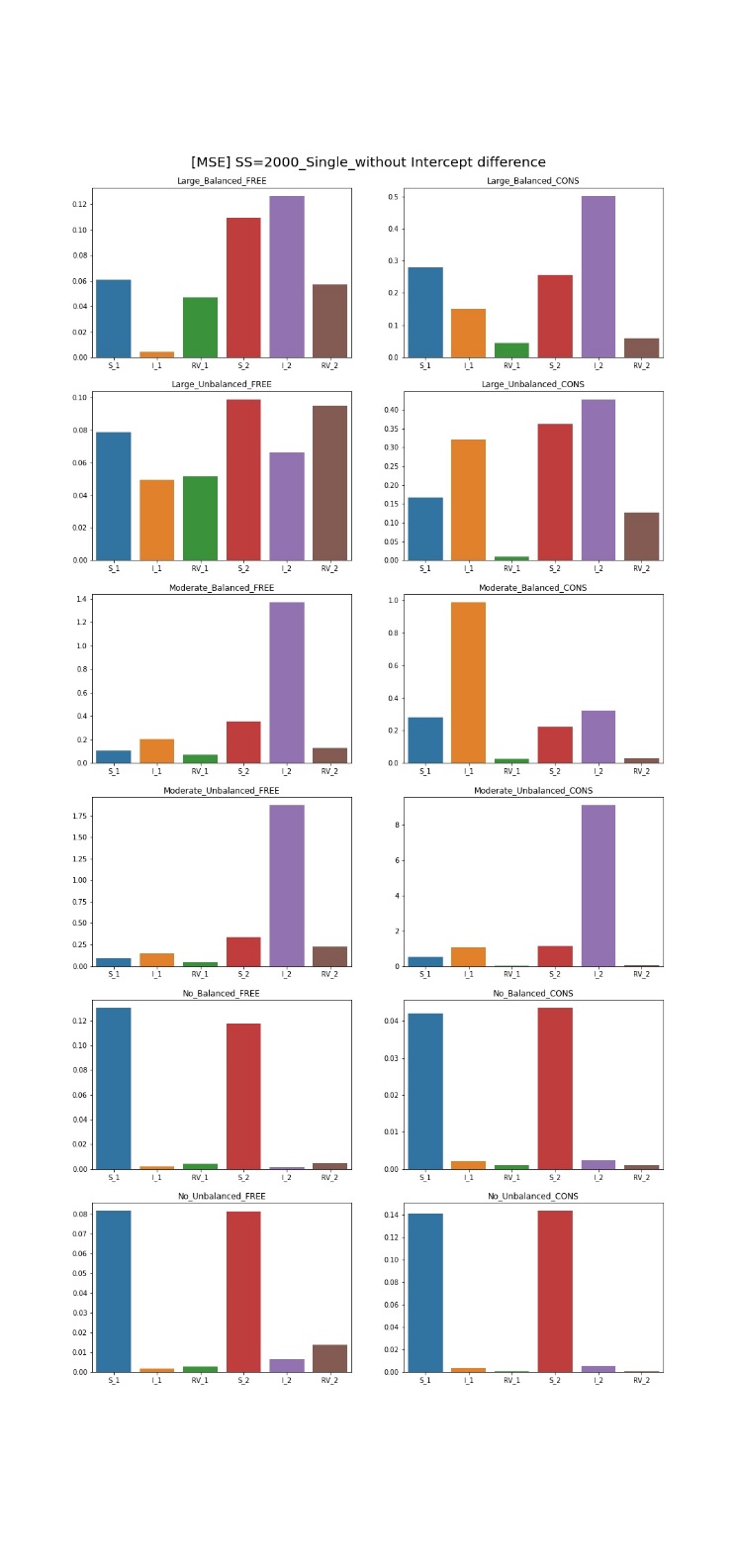


## 2.2 Single-predictor model, with intercept difference


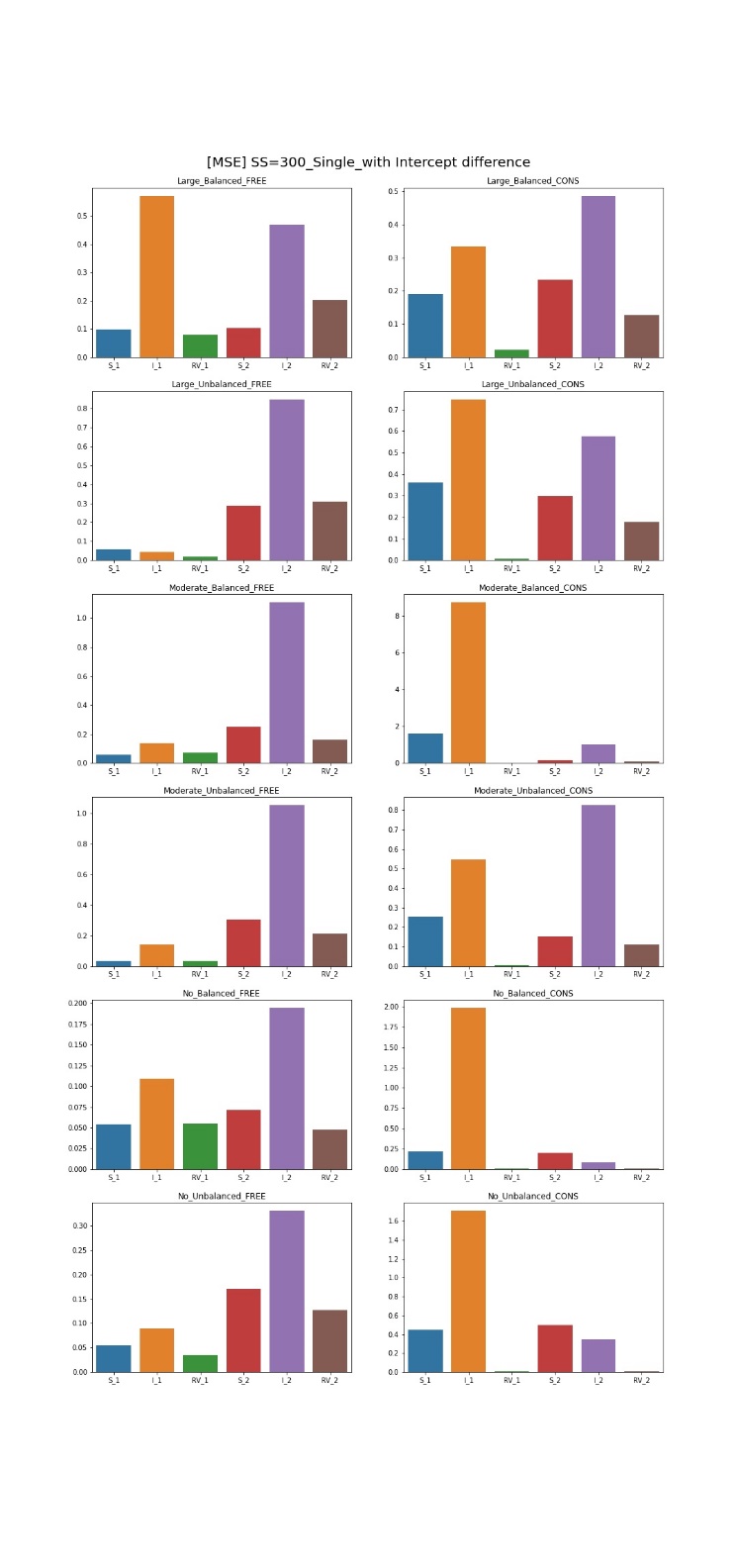

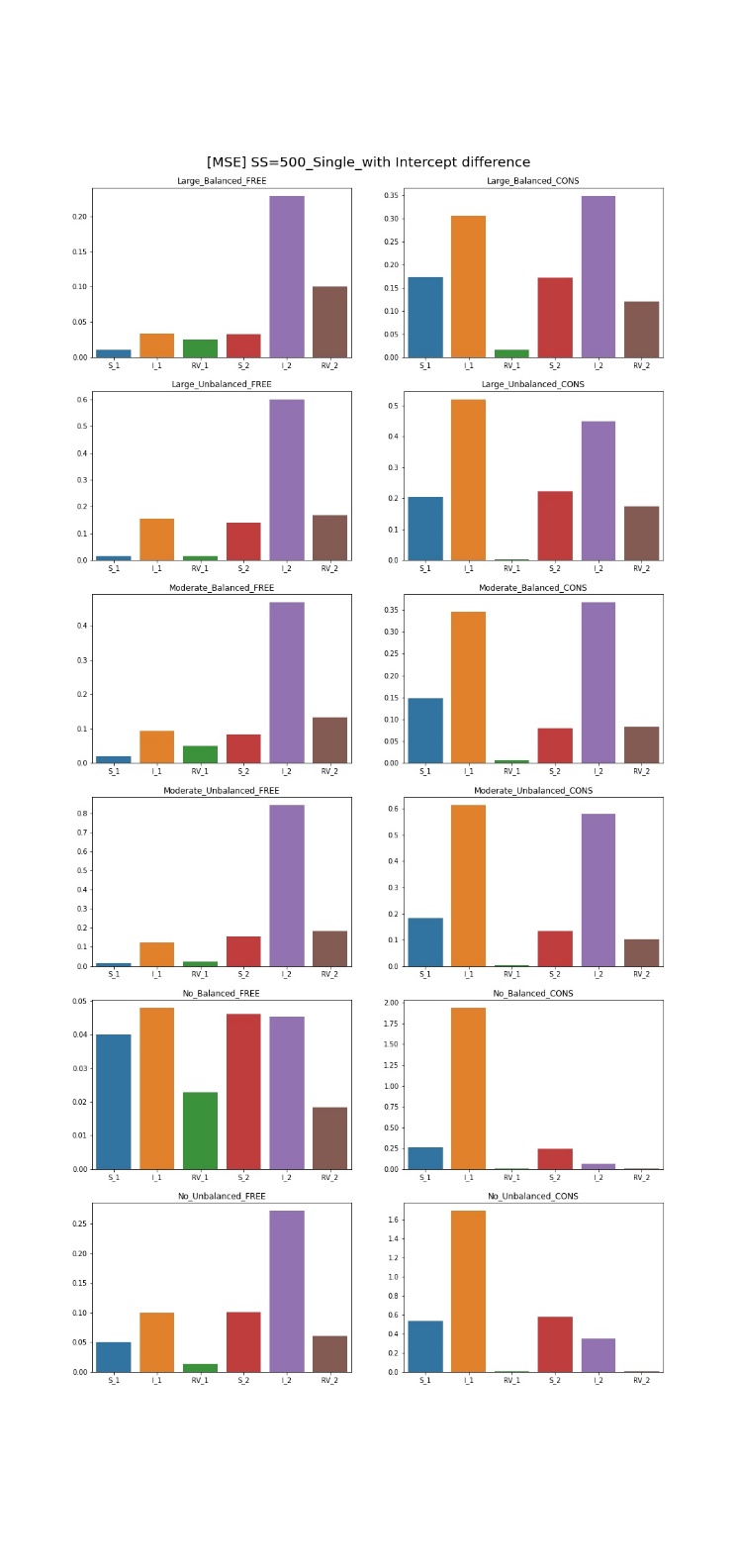


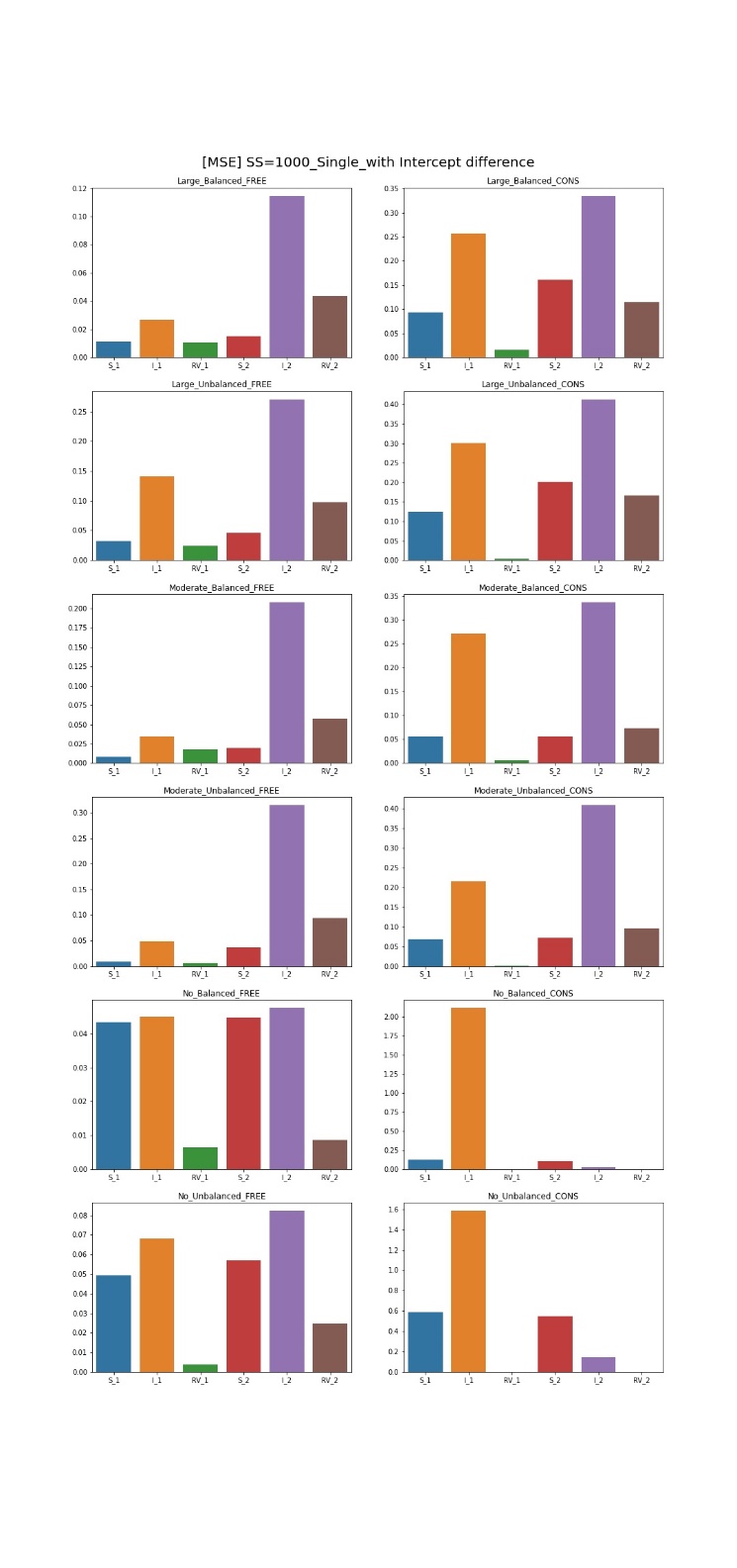

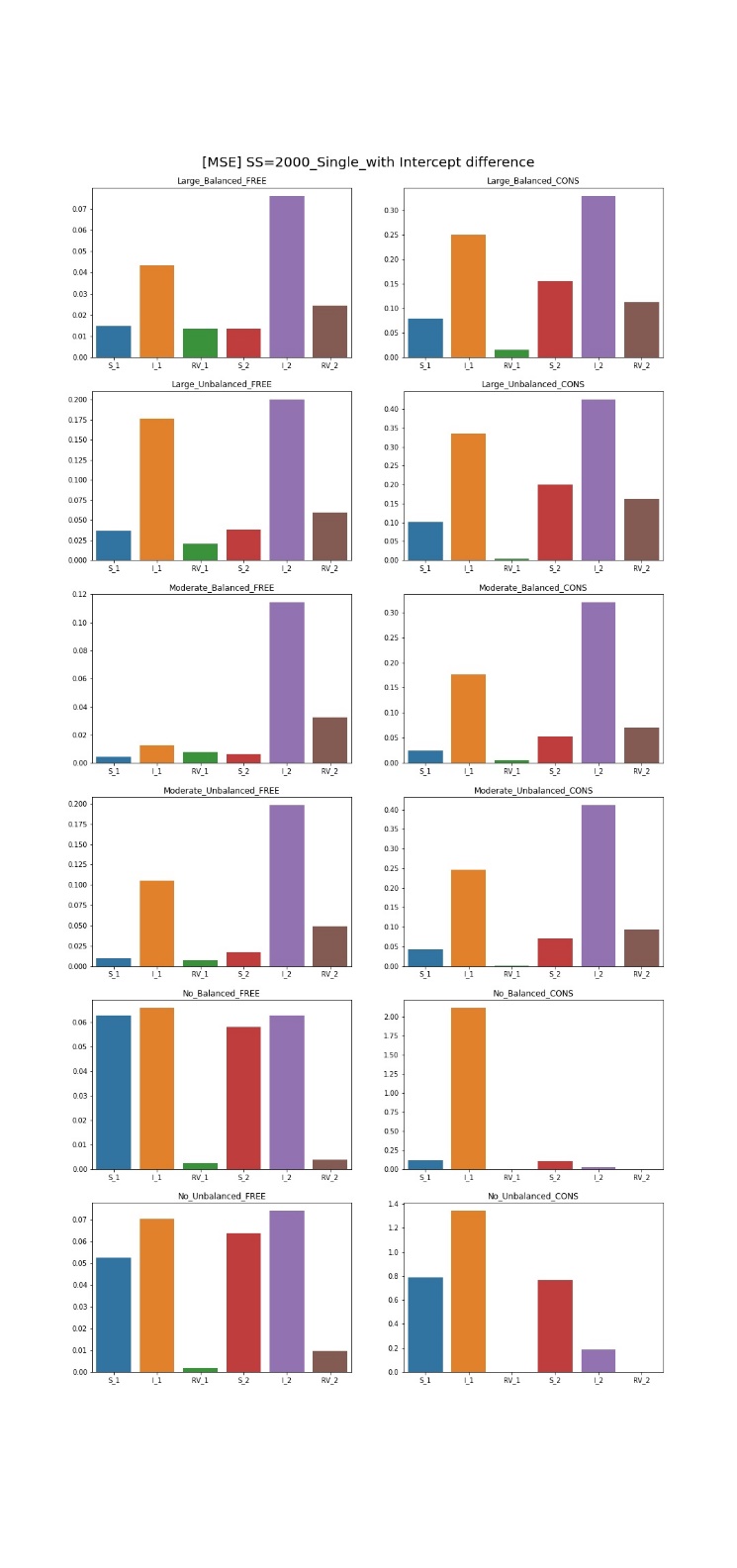


## 2.3 Two-predictor model, without intercept difference


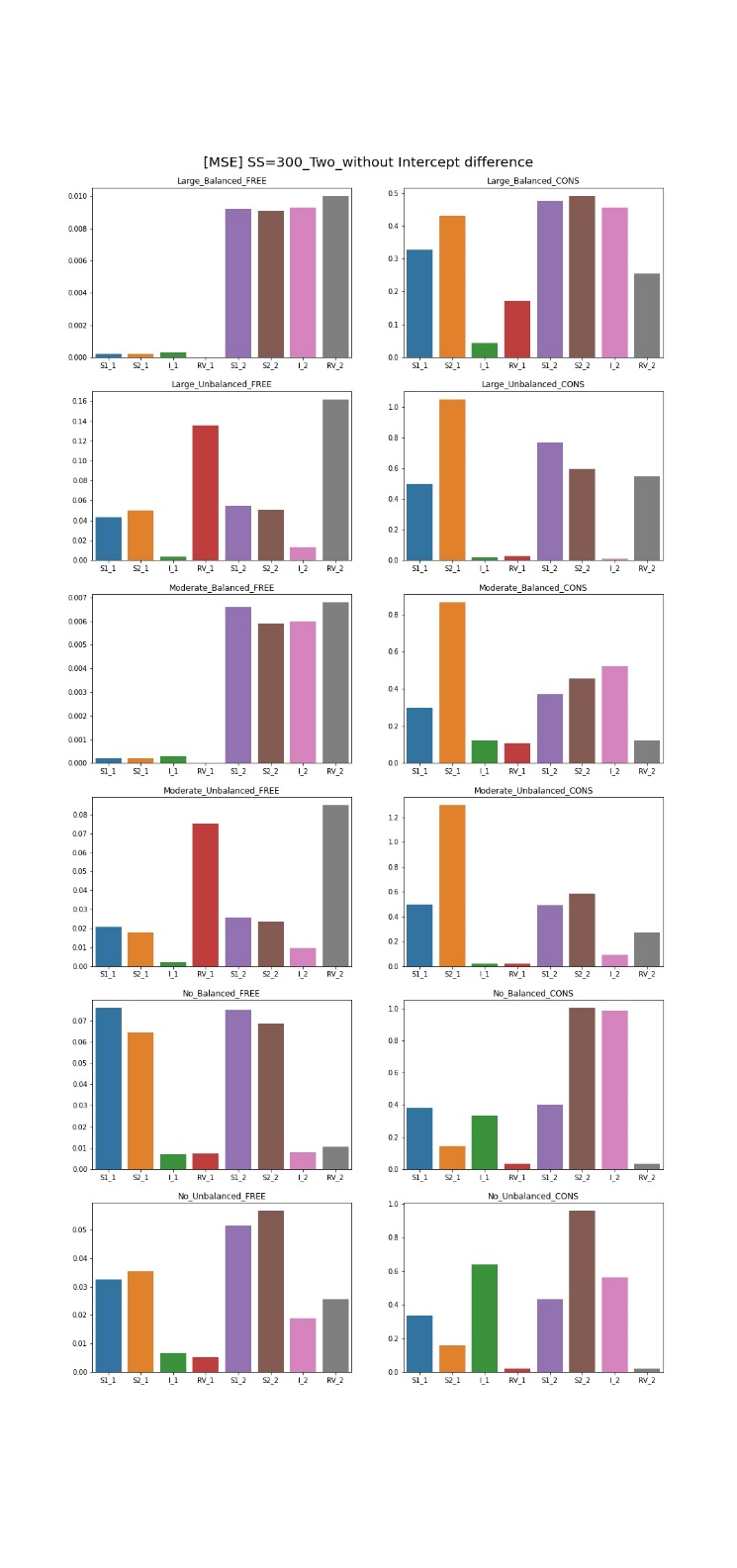


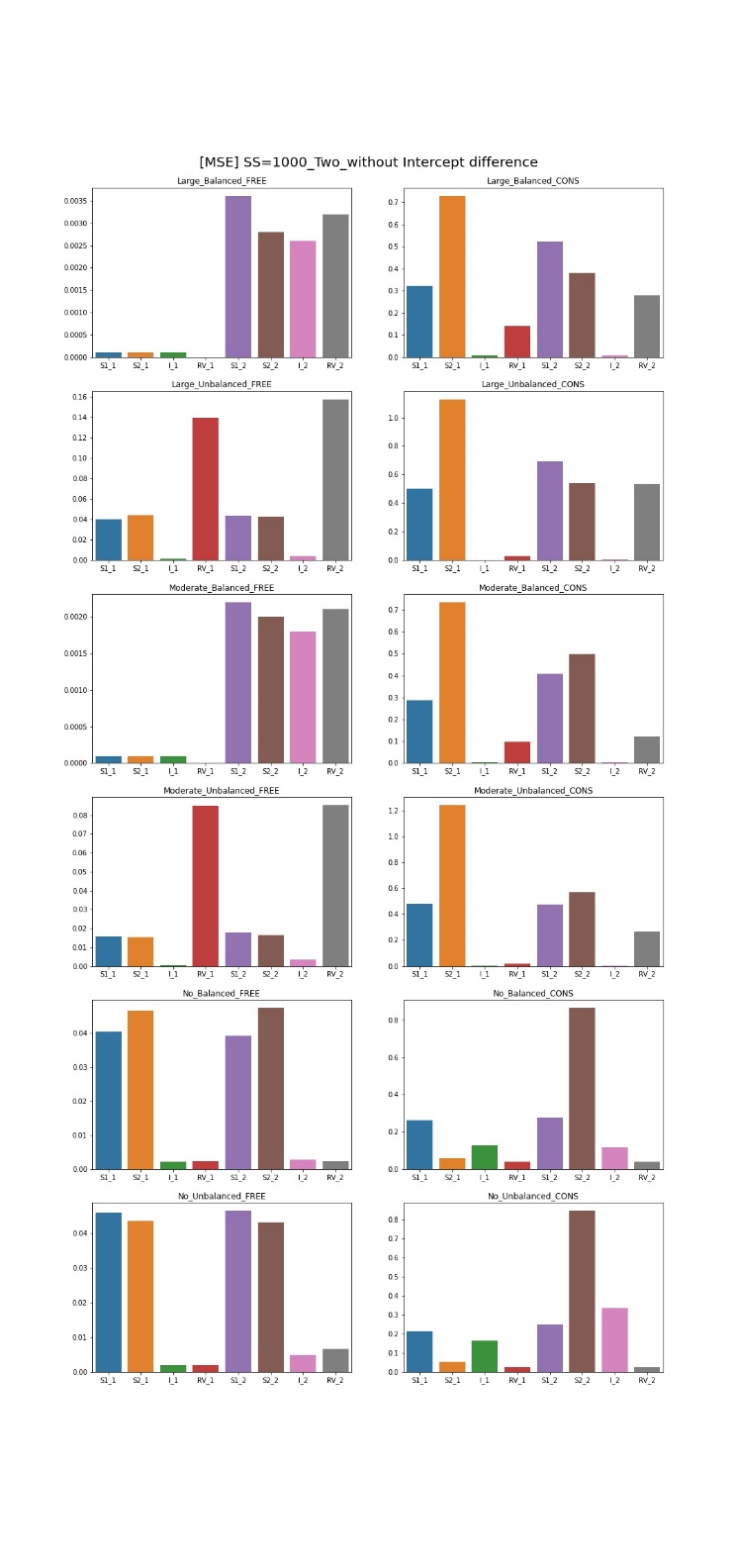

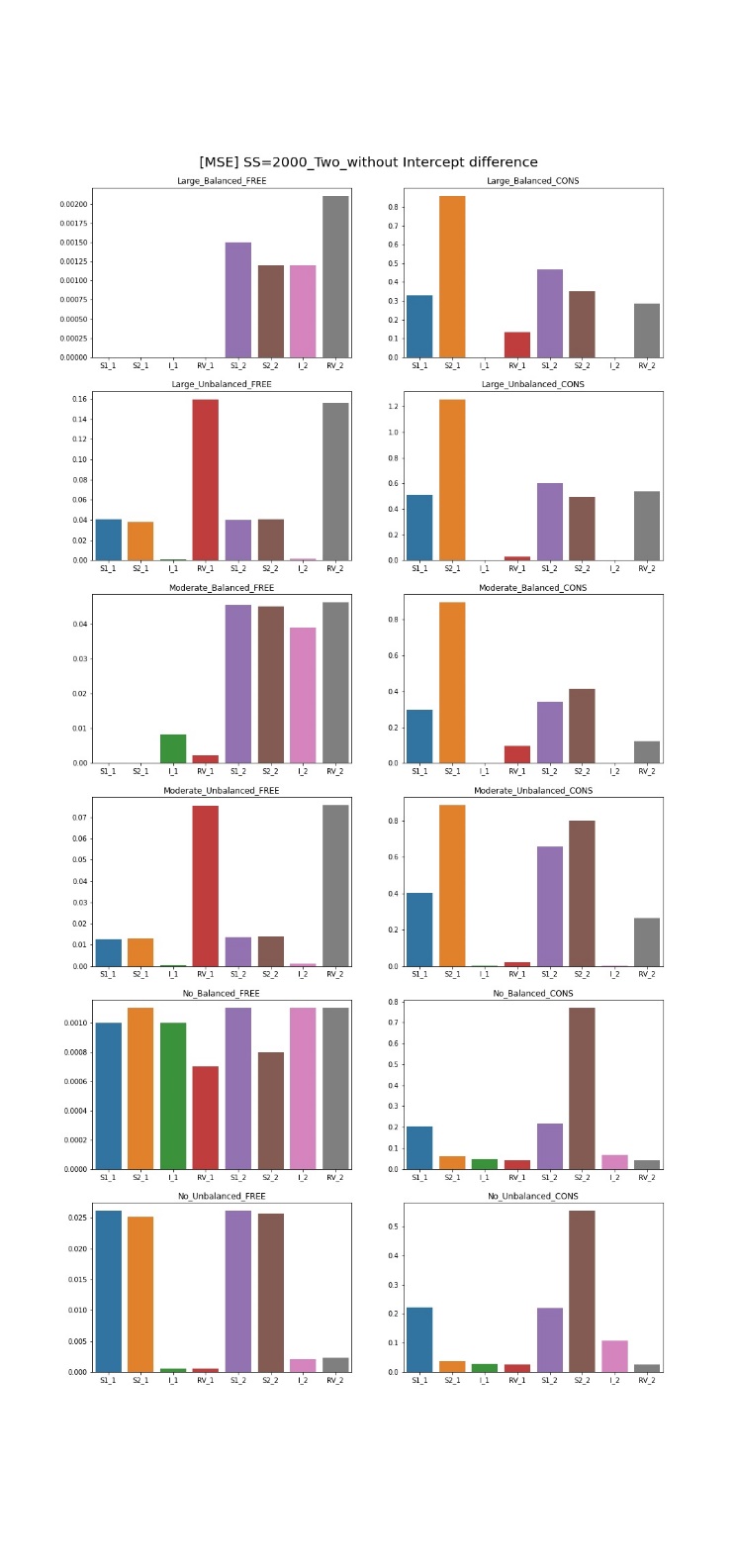


## 2.4 Two-predictor model, with intercept difference


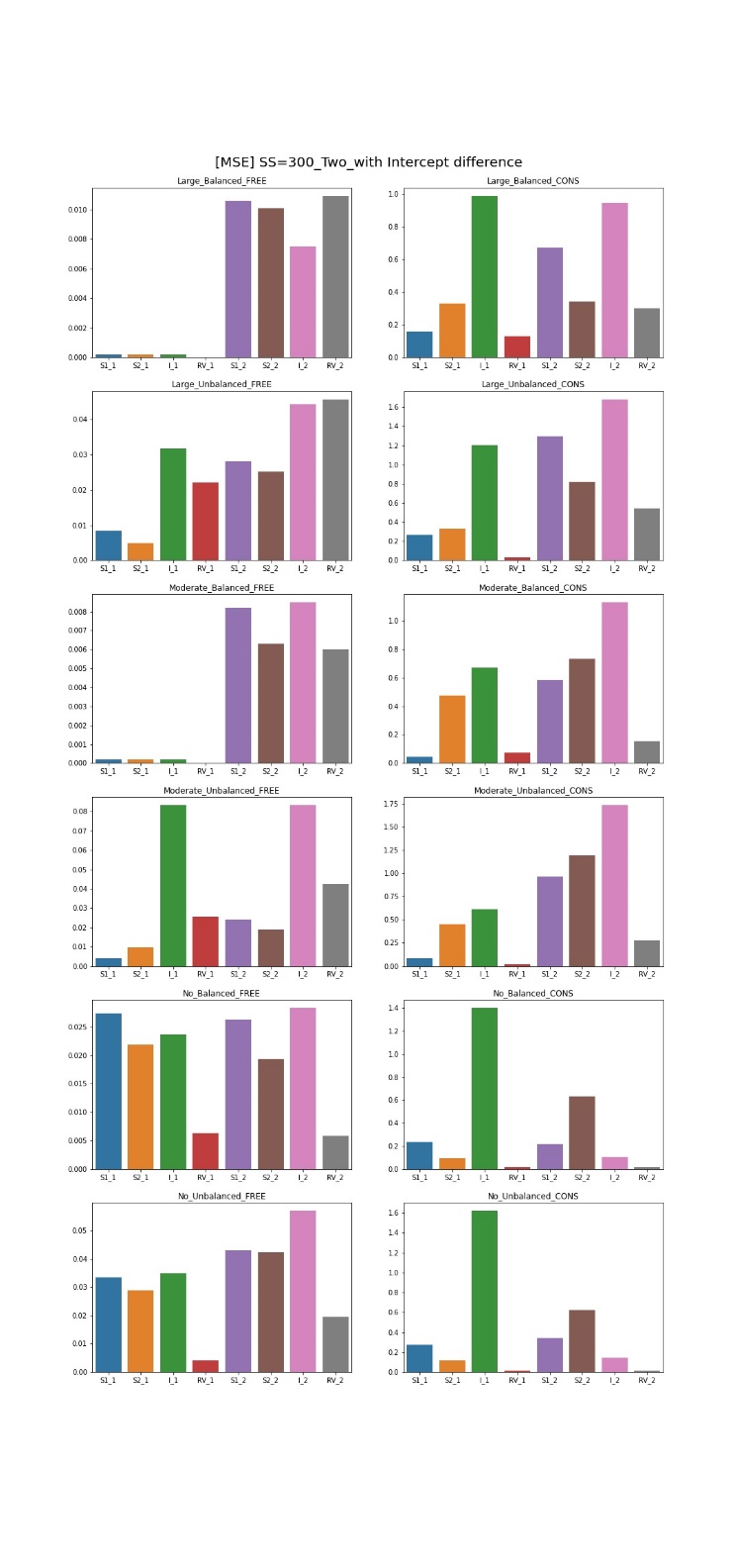

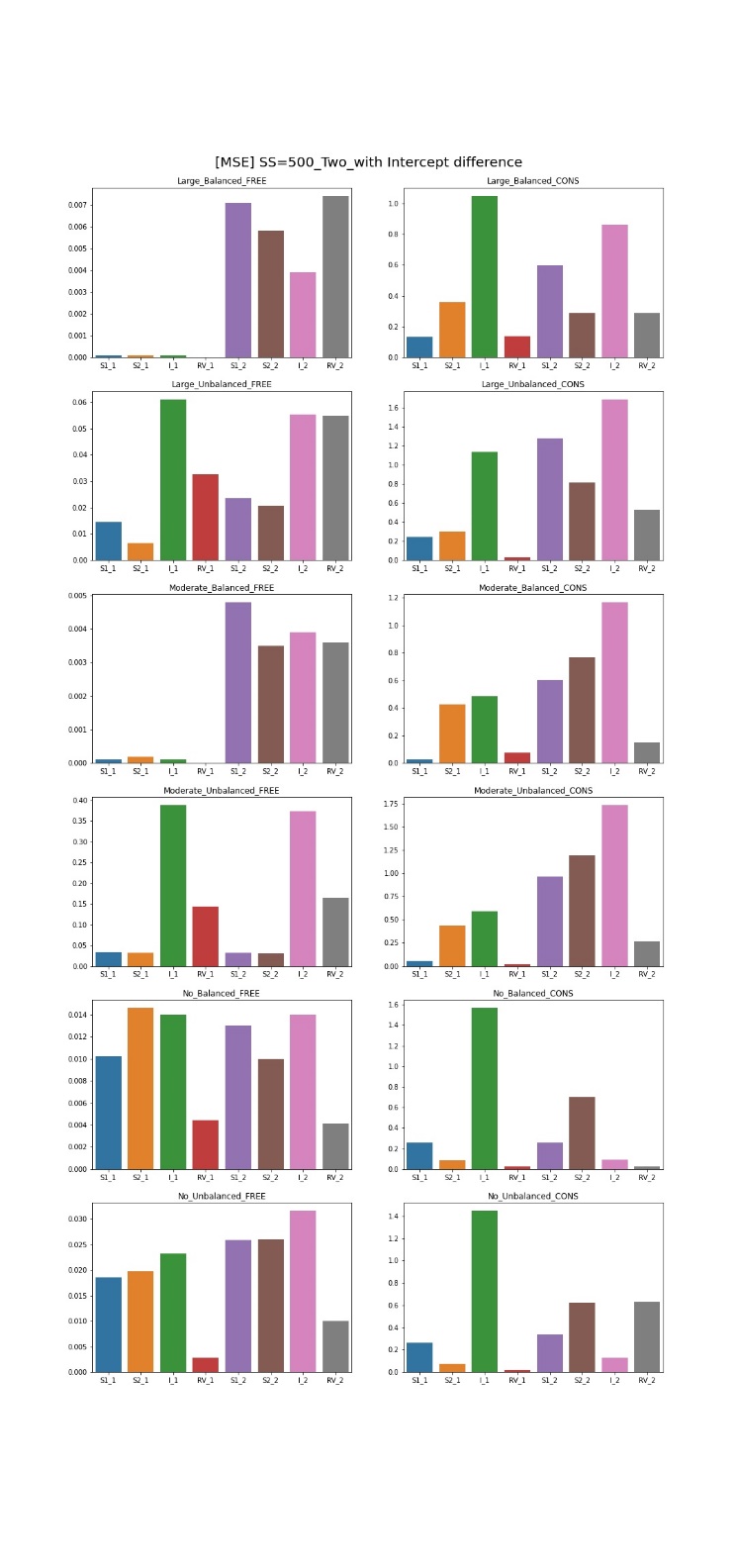


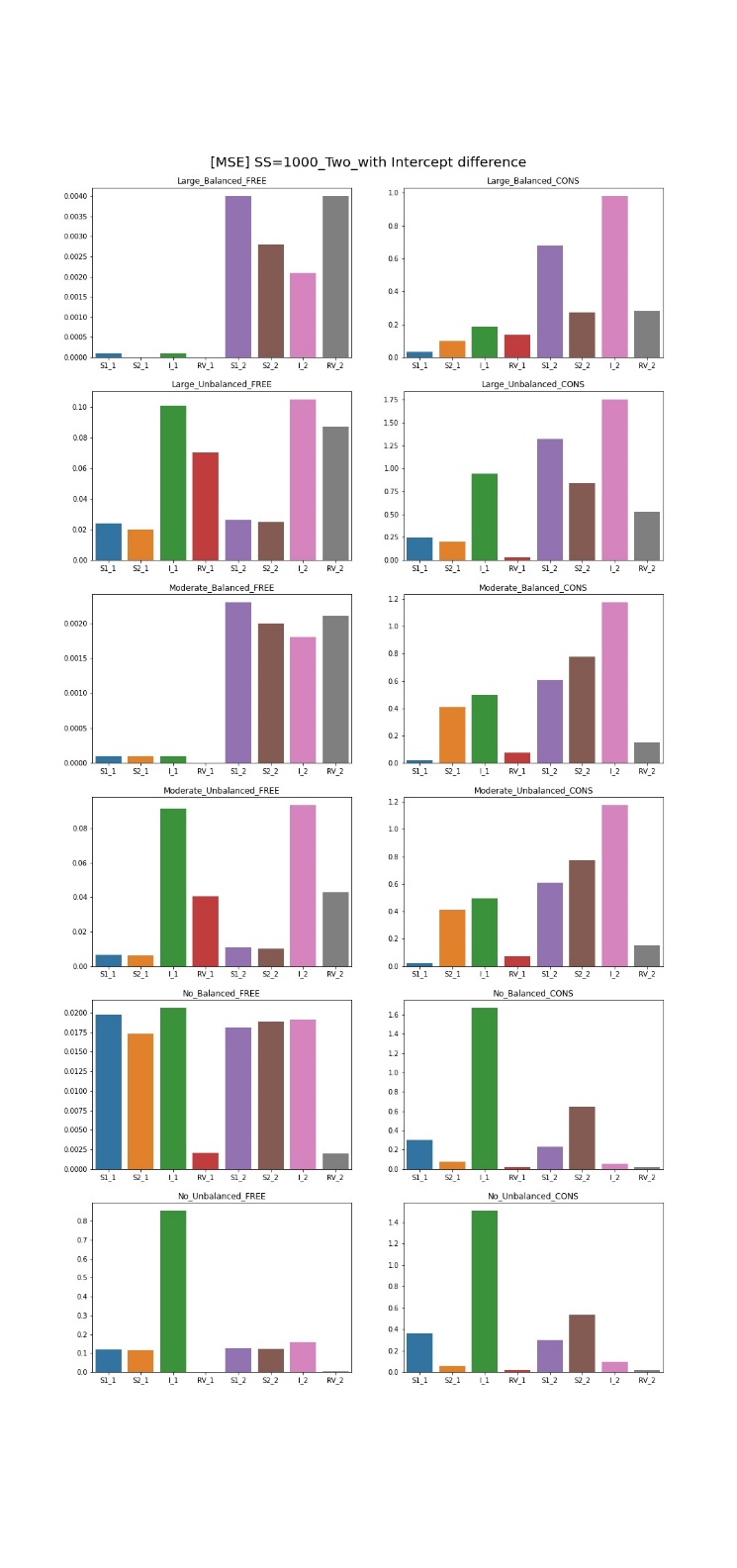

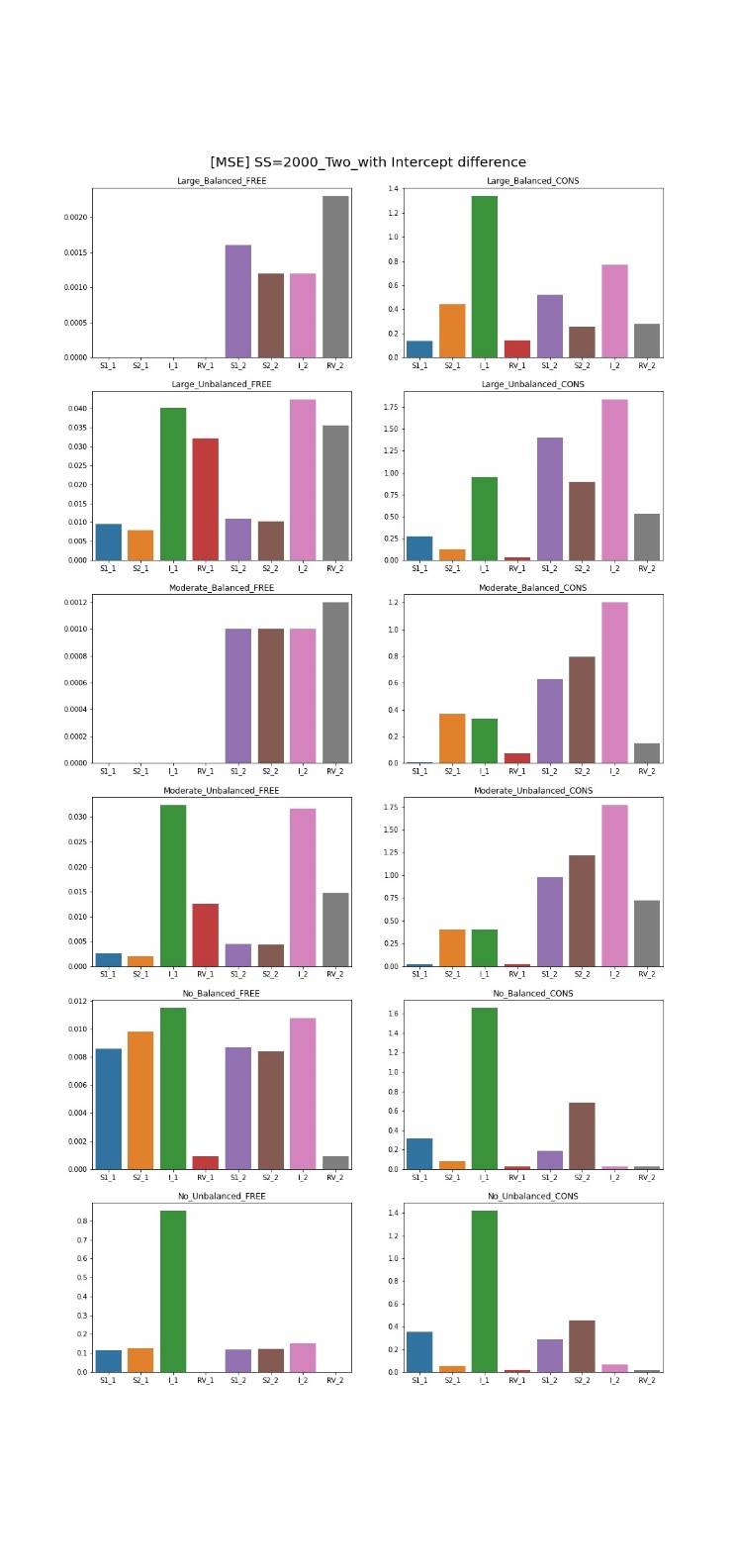


**Coverage**

## 3.1 Single-predictor model, without intercept difference


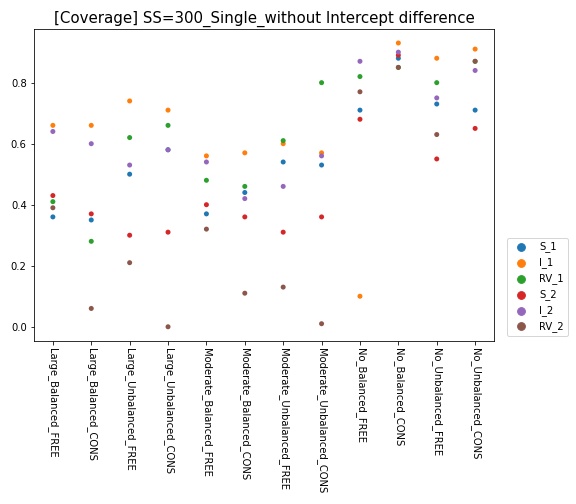


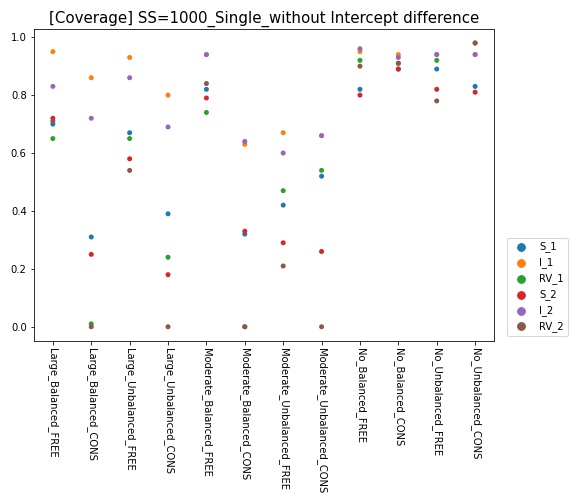

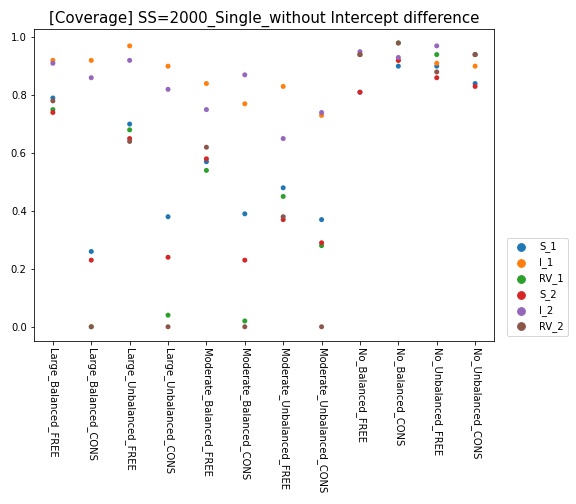


## 3.2 Single-predictor model, with intercept difference


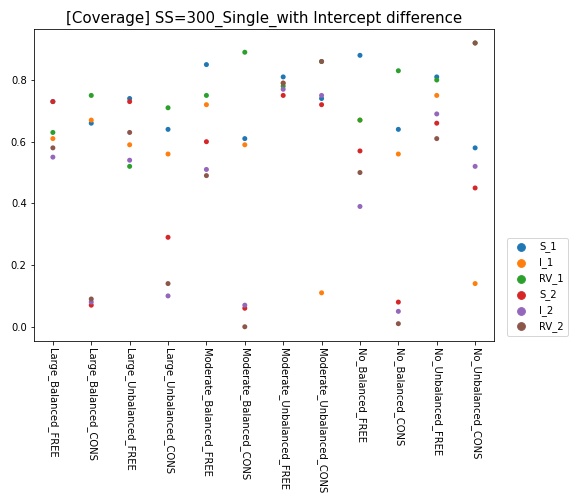

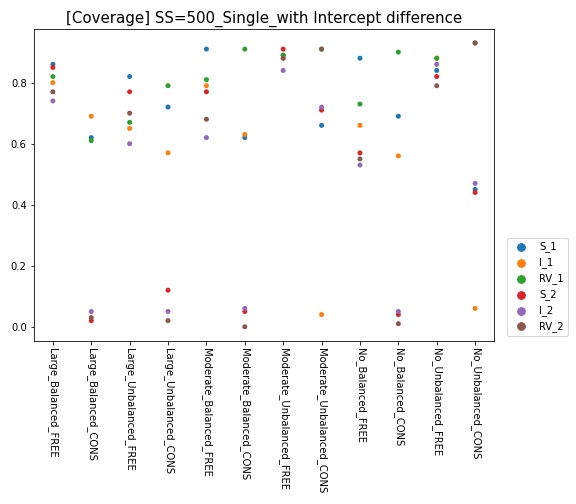


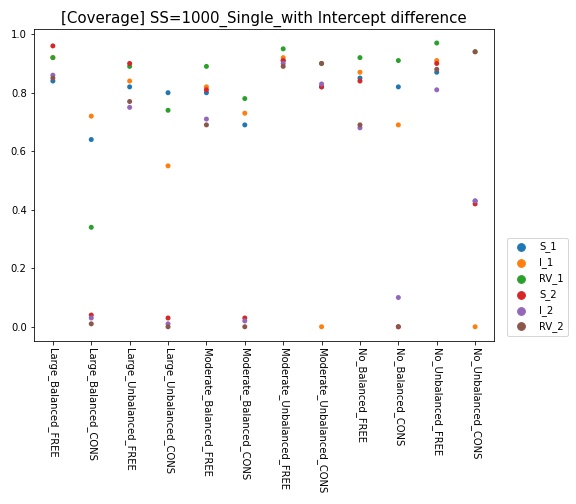

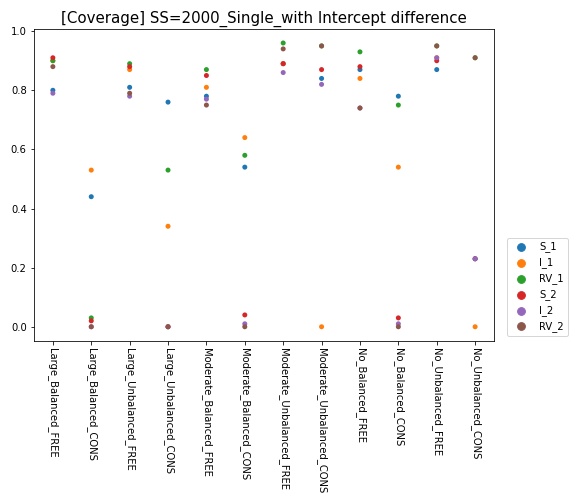


## 3.3 Two-predictor model, without intercept difference


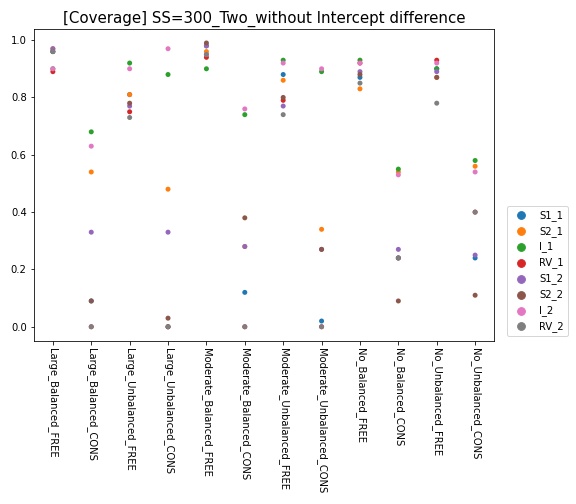


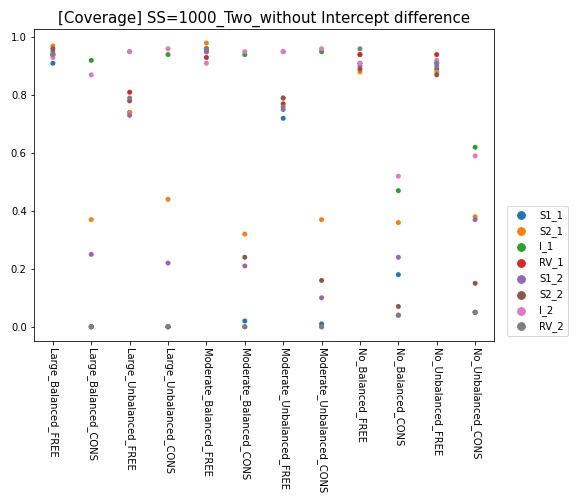

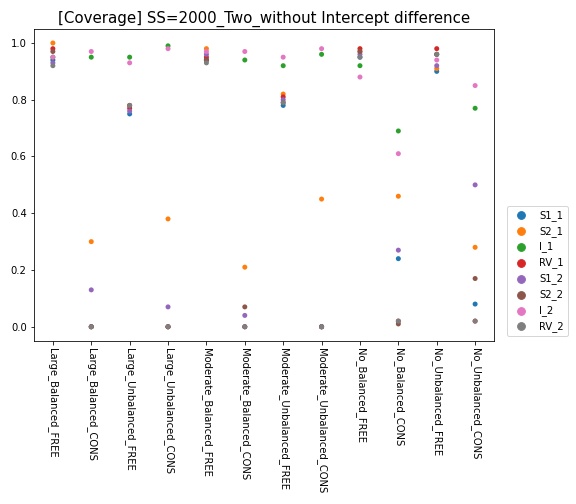


## 3.4 Two-predictor model, with intercept difference


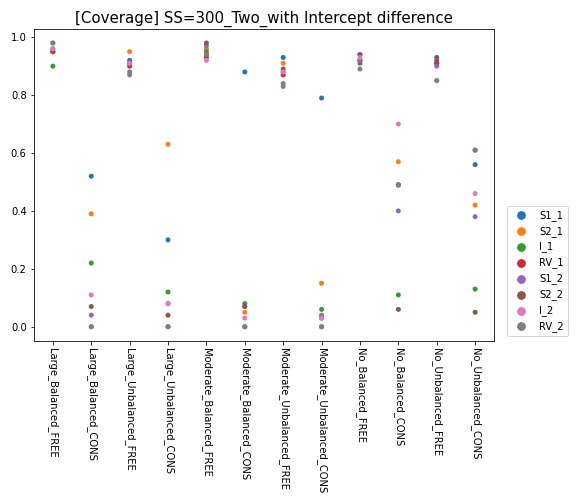

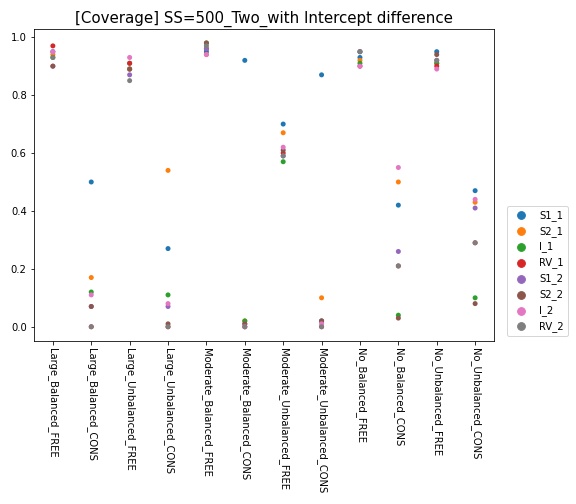


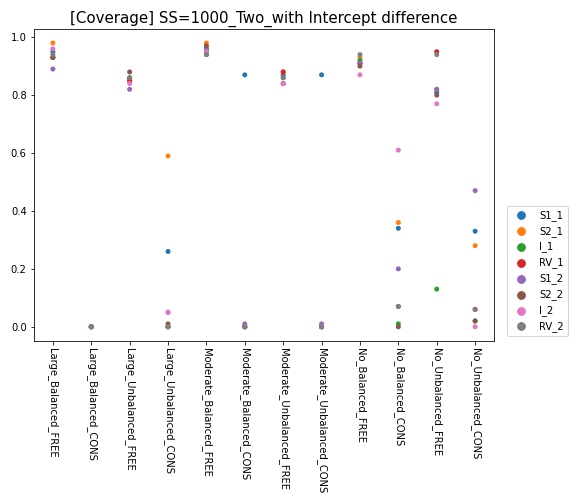

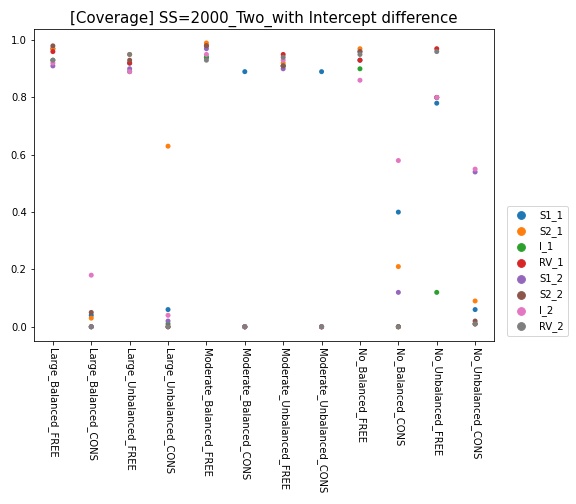

Supplement: Supplementary file 1 [file Data_Sheet_1.docx]
